# Photosynthetic Heat Tolerance Partially Acclimates to Growth Temperature in Tropical Montane Tree Species

**DOI:** 10.1111/pce.70079

**Published:** 2025-07-23

**Authors:** Olivier Jean Leonce Manzi, Myriam Mujawamariya, Lasse Tarvainen, Camille Ziegler, Mats X. Andersson, Mirindi Eric Dusenge, Astrid Fridell, Heather Reese, Cornelia Spetea, Felicien K. Uwizeye, Maria Wittemann, Donat Nsabimana, Göran Wallin, Johan Uddling

**Affiliations:** ^1^ Department of Biological and Environmental Sciences University of Gothenburg Gothenburg Sweden; ^2^ Department of Nature Conservation, Kitabi College Rwanda Polytechnic Huye Rwanda; ^3^ School of Geography University of Leeds Leeds UK; ^4^ Department of Biology, College of Science and Technology University of Rwanda Kigali Rwanda; ^5^ INRAE, UMR BIOGECO University of Bordeaux Pessac France; ^6^ Division of Plant Sciences, Research School of Biology The Australian National University Canberra ACT Australia; ^7^ Department of Earth Sciences University of Gothenburg Gothenburg Sweden; ^8^ School of Agriculture and Food Sciences, College of Agriculture, Animal Sciences and Veterinary Medicine University of Rwanda Musanze Rwanda; ^9^ School of Forestry and Biodiversity Conservation, College of Agriculture, Animal Sciences and Veterinary Medicine University of Rwanda Musanze Rwanda

**Keywords:** chlorophyll fluorescence, elevation gradient, heat stress, leaf temperature, photosynthetic heat tolerance, recovery, thermal safety margin, thylakoid membrane lipids, tropical forest

## Abstract

Climate warming increases the risk of harmful leaf temperatures in terrestrial plants, particularly in tropical tree species that have evolved in warm and thermally stable environments. We examined heat tolerance thresholds of photosynthetic light reactions in sun‐exposed leaves of 12 tropical montane tree species with different strategies for growth and water use. Leaf chlorophyll *a* fluorescence, gas exchange, morphology and thylakoid membrane lipid composition were measured at three common gardens along an elevation and temperature gradient in Rwanda. Tree species with traits predisposing them to higher leaf temperatures, such as lower stomatal conductance and large leaves, had higher photosynthetic heat tolerance, but narrower thermal safety margins (TSMs). Photosynthetic heat tolerance partially acclimated to increased growth temperature, increasing by 0.31°C on average for every 1°C increase in growth temperature. Thus, TSMs were narrower for trees grown at the warmer sites. Heat tolerance and its acclimation were linked to the adjustment of thylakoid membrane lipid composition. Moreover, TSMs were larger in species with high leaf mass per area. Our results show that (i) leaf temperature is more important than heat tolerance in controlling interspecific variation in TSMs, and that (ii) tropical trees have limited ability to thermally acclimate to increasing temperatures.

## Summary Statement

Climate warming poses a risk to tropical montane trees by narrowing their thermal safety margins, particularly in species with traits that lead to higher leaf temperatures. While photosynthetic heat tolerance shows some acclimation to warmer growth conditions, the limited extent of this plasticity suggests tropical trees may struggle to keep pace with rising temperatures.

## 1 Introduction

Tropical forests are threatened by climate change. Projections indicate a continued rise in temperatures accompanied by more frequent and severe heat waves (IPCC 2021). Trees will thus be exposed to temperatures above their upper photosynthetic heat tolerance (PHT) more often, causing declines in performance and competitiveness (Feeley et al. 2011; Doughty et al. 2023; Li et al. 2024). Such shifts may induce subsequent alterations in tree community composition, favouring warm‐adapted species over cold‐adapted, that is, thermophilisation (Duque et al. 2015; Fadrique et al. 2018; Esquivel‐Muelbert et al. 2019; Ntirugulirwa et al. 2023; Cuni‐Sanchez et al. 2024). Given that ongoing anthropogenic climate change is too fast for most long‐lived species to respond adequately through migration and evolutionary adaptation, the competitiveness of tropical trees and the composition of tropical forests will depend on the ability of individuals to acclimate to rising temperatures (Feeley et al. 2023).

In response to the growing concern regarding the potential impact of heat stress on forest ecosystems, there has been an increase in scientific studies exploring the photosynthetic heat sensitivity of different plant species. These have revealed that plants deal with heat through avoidance as well as tolerance mechanisms, both of which serve to increase the plant's thermal safety margin (TSM). The TSM can be defined as the difference between the maximum leaf temperature (*T*_leafmax_) and the PHT threshold beyond which photosynthesis is impaired (Mathur et al. 2014; Teskey et al. 2015; Cook et al. 2021; Kitudom et al. 2022; Tarvainen et al. 2022). Avoidance mechanisms include adjusting leaf thermoregulatory traits such as developing smaller leaves or more reflective leaf surfaces (Leigh et al. 2017; Wright et al. 2017), adjusting leaf angles to reduce radiation absorption or enhancing leaf cooling through increased transpiration (Aparecido et al. 2020; Tarvainen et al. 2022; Zhou et al. 2023; Posch et al. 2024). These thermoregulatory traits contribute to maintenance of leaf temperatures within the optimal range for photosynthesis and widen the TSM to avoid or minimise heat stress (Michaletz et al. 2015; Fauset et al. 2018; Drake et al. 2020; Moran et al. 2023). However, recent studies have shown that tropical trees exhibit limited ability for thermoregulation, with sun‐exposed outer canopy leaves experiencing temperatures up to 15°C–20°C above the ambient air temperature (Fauset et al. 2018; Crous et al. 2023; Manzi et al. 2024; Javad et al. 2025). With the limited ability for heat avoidance, trees also rely on adjusting biochemical traits that increase their PHT to cope with heat stress (Krause et al. 2010; Slot et al. 2021).

Studies on co‐occurring species have shown that PHT may differ substantially between tree species growing under similar conditions (Zhang et al. 2012; Curtis et al. 2019; Perez and Feeley 2020; Perez et al. 2021; Tarvainen et al. 2022; Okubo et al. 2023). These interspecific differences are likely linked to both biochemical and morphological adaptations. Biochemical analyses have identified various factors enhancing PHT, such as the increased production of osmolytes which stabilise proteins (Hüve et al. 2011; Posch et al. 2024), modifications in thylakoid membrane lipid composition to maintain optimal membrane fluidity (Zhu et al. 2018, 2024; Tarvainen et al. 2022), elevated emissions of biogenic volatile compounds (Taylor et al. 2019; Liu et al. 2021), and enhanced expression of heat shock proteins to mitigate damage to proteins (Aspinwall et al. 2019; Bakery et al. 2024). Despite the considerable variation in PHT among species (O'sullivan et al. 2017; Feeley et al. 2020; Slot et al. 2021), little is known regarding the relative importance of different biochemical mechanisms leading to this variation under field conditions, particularly in tropical forests.

In addition to biochemical adaptations, morphological traits may be linked to heat tolerance. Some studies have indicated that species with higher leaf mass per area (LMA) tend to exhibit greater physiological heat tolerance (e.g., Sastry and Barua 2017; Zhang et al. 2024). This suggests that leaves with higher construction costs are better adapted to withstand extreme heat than their less costly counterparts, in line with general expectations on conservative and stress‐tolerant species (Wright et al. 2004). Leaves with higher LMA typically possess thicker mesophyll layers, denser cell packing, and greater investment in protective compounds (e.g., heat shock proteins, antioxidants), which collectively can enhance leaf thermal stability and resistance to heat‐induced cellular damage (Poorter et al. 2009; O'sullivan et al. 2017). In addition, high LMA is often associated with higher leaf heat capacity, allowing such leaves to buffer more effectively against rapid temperature fluctuations and short extreme heat events (Vogel 2009; Slot et al. 2021). However, empirical support for the link between LMA and PHT remains mixed, with some studies reporting positive associations (Sastry and Barua 2017; Sastry et al. 2018; Slot et al. 2021; Li et al. 2022; Zhang et al. 2024), while others found no significant relationship (Fadrique et al. 2022; Münchinger et al. 2023; Bison and Michaletz 2024).

While the threats of climate change to tropical forests are widely recognised, and physiological acclimation may have a significant role in countering these effects, our knowledge of the acclimation of PHT to warming in tropical trees is derived from a limited number of studies conducted in controlled environments (e.g., Zhu et al. 2018, 2024; Cox et al. 2025). Existing field studies have explored spatial PHT variation along natural environmental gradients (Feeley et al. 2020; Araújo et al. 2021; Slot et al. 2021; Kullberg et al. 2024) or temporal variation between seasons (Sastry et al. 2018; Zhu et al. 2018; Tiwari et al. 2021; Kullberg and Feeley 2024). However, none of these approaches is ideal for determining the acclimation capacity of tree species. The environmental gradient studies compared different species or ecotypes present at different locations, complicating separation between acclimation and local adaptation, and seasonal shifts may be influenced by non‐thermal factors such as phenology or the co‐variation in soil water availability.

To our knowledge, only one non‐enclosure field study has investigated the acclimation of PHT in tropical trees without confounding influences of local genetic adaptation (Tarvainen et al. 2022). That study used three tropical montane tree species with different growth and water‐use strategies, planted and grown at different sites along an elevation gradient in Rwanda. It indicated that PHT may partially acclimate to warming and that species with bigger leaves and lower stomatal conductance (*g*_s_) exhibited higher *T*_leaf_ and higher PHT. However, it is hard to draw general conclusions from this single study since it included only three species. Moreover, it relied on modelled *T*_leaf_, potentially leading to inaccuracies in estimating *T*_leaf_ and, thus, TSM. There is therefore a critical need for additional in‐situ experiments with a broad range of species and *T*_leaf_ observations to better assess heat effects on photosynthesis in a warming climate.

Utilising multi‐species plantations along a natural elevation and temperature gradient in Rwanda TRopical Elevational Gradient Experiment (TREE) project, we investigated the acclimation potential of PHTs and TSMs to warming in 12 tropical tree species and to what extent it was linked to leaf biochemical, physiological and morphological traits. The Rwanda TREE project comprises three sites with large variation in elevation (1300–2400 m above sea level) and temperature (17.1°C–24.0°C mean daytime temperature). The plantations were established using seedlings propagated from common seed material, enabling us to investigate both interspecific variation in PHT and its potential to acclimate to warming. By controlling for genetic differences, our study avoids confounding adaptation with acclimation responses. In addition, direct measurement of *T*_leaf_ in this study further enhances the accuracy of TSM estimates compared to using *T*_air_. Specifically, we evaluated the following predictions:

1. Tree species with traits that predispose them to high leaf temperatures (i.e., large leaves and lower *g*_s_) also have higher PHT, but not high enough to prevent a narrowing of TSM.
2. Tree species exhibit partial acclimation to rising growth temperatures, with *T*_leaf_ increasing faster than PHT, resulting in narrower TSMs under warmer climate.
3. Warming‐induced shifts in PHT are linked to adjustments in leaf osmolality and thylakoid membrane lipid composition, such that an increase in leaf osmolality and/or decrease in membrane fluidity leads to higher PHT.
4. PHT and TSM are linked to leaf construction cost, such that species with higher LMA exhibit higher PHT and wider TSM.

## 2 Materials and Methods

### 2.1 Field Sites and Plant Material

This study was conducted at three field sites within the Rwanda TREE project (www.rwandatree.com). Sigira (2°30′54″ S, 29°23′44″ E), the highest‐elevation (HE, 2400 m.a.s.l.) site, has cool and wet conditions with an annual mean daytime temperature of 17.5°C and 2100 mm of annual precipitation. Rubona (2°28′30″ S, 29°46′49″ E), the mid‐elevation (ME, 1600 m.a.s.l.) site, is warmer and drier, with a mean daytime temperature of 22.5°C and annual precipitation of 1600 mm. Makera (2°6′31″ S, 30°51′16″ E), the lowest elevation (LE, 1300 m.a.s.l.) site, is the warmest and driest, with a mean daytime temperature of 23.8°C and annual precipitation of 1050 mm. The rainfall is highest from March to May, followed by a dry period from June to August, sometimes extending into September at the lower sites.

Each field site consists of 1800 trees from 20 species native to and common in Rwanda, planted into 18 quadratic plots with a total of 100 trees in each (i.e., five individuals per species). The trees were planted randomly with a spacing of 1.5 m in December 2017–January 2018. The 18 plots allowed for a full factorial experimental design with three water levels and two fertility levels (fertilised and unfertilised) and a replication of three plots for each of the six treatment combinations. The three water levels were achieved through irrigation and throughfall exclusion treatments, reproducing the rainfall amounts that occur naturally at the three sites, resulting in all sites having the same three water input levels (precipitation ± manipulation). Fertilisation of N/P/K/Mg/S/Zn was 100/68/55/5.0/7.5/0.75 kg ha^−1^ in February–March 2021 and March–April 2022 and half of these amounts in November 2019. On the same occasions, the fertilised plots were limed with 2500 kg ha^−1^ in the HE site, and a fifth of this amount in the ME site, to compensate for soil pH differences between sites. For more detailed information on the experimental sites and design, see Ntirugulirwa et al. (2023). The 12 species used in this study were selected to represent different successional strategies and climate origins (Table 1), and to span a broad spectrum of traits including leaf size (5–228 cm²), LMA (65–147 g m⁻²; Manishimwe et al. 2022), and peak daytime stomatal conductance (g_s_; 0.08–0.40 mol m⁻² s⁻¹; Mujawamariya et al. 2023).

**Table 1:** Tree species included in this study with information on their successional identities, abbreviation codes, taxonomic families and elevation ranges.

Successional group	Scientific name	Code	Family	Elevation range (m.a.s.l)^c^
Early successional	*Croton megalocarpus* Hutch.	*Cme*	Euphorbiaceae	700–2400
	*Harungana montana* a Spirlet	*Hmo*	Hypericaceae	1950–2500
	*Macaranga kilimandscharica* Pax	*Mki*	Euphorbiaceae	1700–2700
	*Maesa lanceolata* Forssk.	*Mla*	Primulaceae	1350–3000
	*Markhamia lutea* (Benth.) K. Schum.	*Mlu*	Bignoniaceae	700–2000
	*Polyscias fulva* (Hiern) Harm	*Pfu*	Araliaceae	1700–2900
Late successional	*Afrocarpus falcatus* (Thunb.) C. N. Page	*Afa*	Podocarpaceae	1800–3000
	*Carapa grandiflora* Sprague	*Cgr*	Meliaceae	1625–2525
	*Entandrophragma excelsum* (Dawe & Sprague)^a^	*Eex*	Meliaceae	1500–2150
	*Faurea saligna* Harv.	*Fsa*	Proteaceae	1575–2475
	*Prunus africana* (Hook.f.) Kalkman^b^	*Paf*	Rosaceae	1600‐3200
	*Syzygium guineense* (Willd.) DC.^a^	*Sgu*	Myrtaceae	1350–2700
Mixed successional	*Ficus thonningii* Blume	*Fth*	Moraceae	1000–2500

^a^ Heat tolerance data for these species were previously published by Tarvainen et al. (2022).

^b^ This species was only measured at the mid‐elevation site and in the lab.

^c^ Commonly observed natural elevation ranges in the region of the study (Ntirugulirwa et al. 2023).

### 2.2 Chlorophyll Fluorescence Measurements in the Field

The variation in PHT among species and sites was investigated using chlorophyll fluorescence methodology (Maxwell and Johnson 2000; Murchie and Lawson 2013) in February and March 2021. This approach evaluates the temperature response of photosystem II (PSII) activity by measuring either the minimum value of chlorophyll fluorescence (minimum fluorescence yield in the dark state, *F*_0_), which indicates the number of open reaction centres, or the dark‐acclimated maximum quantum yield of PSII photochemistry (*F*_v_/*F*_m_). Here, *F*_m_ refers to maximum fluorescence yield in the dark state, representing chlorophyll fluorescence under a short saturating light pulse when all reaction centres are closed, and *F*_v_ refers to variable fluorescence (*F*_v_) which is the difference between *F*_m_ and *F*_0_.

In early studies using chlorophyll fluorescence, the *F*_0_ fluorescence parameter was employed to quantify heat tolerance of PSII activity. However, *F*_0_ can provide biased estimates of PSII function during heat treatments due to heat‐induced changes in the leaf optical properties (Baker 2008). Consequently, to assess PHT thresholds in this study, we exclusively used *F*_v_/*F*_m_ which is an overall indicator of photosynthetic performance. The PHT thresholds were determined by fitting this equation describing the relationship between leaf temperature (*x*‐axis) and *F*_v_/*F*_m_:

(1) $$y=\frac{\theta_{a}}{1-e^{-(\theta_{b}+\theta_{C}T)}}$$

where *θ*_a_ is the asymptote of the relationship, *θ*_b_ is a constant and *θ*_c_ is the decay parameter and *T* is the leaf temperature (Perez et al. 2021). Three different thresholds were calculated: *T*_crit_, defined as the temperature at which the slope of the *F*_v_/*F*_m_ versus temperature relationship reached 15% of its steepest value, and *T*_50_ and *T*_95_, representing the temperatures causing 50% and 95% reduction in *F*_v_/*F*_m_ compared to the unstressed value measured at 20°C–25°C (0.803 ± 0.04), respectively.

At each site, we collected 42 mature, healthy and sun‐exposed leaves from each species (6 leaves × 7 target temperatures). These leaves were taken from trees with sun‐exposed branches growing in the three control plots (i.e., no water or nutrient treatment), with 3–4 leaves per tree being collected from 12 to 15 trees per species (3 plots by 5 replicate trees = 15 trees if all alive and sun‐exposed). For each species and site, six different leaves were measured at each of the seven target temperatures. The measurement temperature range spanned from 20°C to 50°C and included seven target temperatures (34°C, 38°C, 42°C, 44°C, 46°C, 48°C and 50°C) as well as ambient room temperature at 20°C–25°C, which was measured before heat treatment in all leaves. This temperature range was selected based on previous findings in Rwanda TREE, which showed that 50°C was sufficient to capture the full *F*_v_/*F*_m_ response of similar tropical montane tree species (Tarvainen et al. 2022). All fluorescence response curves were visually inspected, and none were excluded, as all had declined to zero or near‐zero *F*_v_/*F*_m_ before 50°C (Figure S2). The leaves were collected using pruners attached to a pole and were stored in plastic bags placed inside a cooling box (18°C–20°C throughout the day) when not measured. Wet tissue papers were placed inside the bags to avoid leaf dehydration. We sampled and measured one species per day. To avoid that no prior heat stress had occurred on the sampling day, the sampling was done predawn. Temperatures on the days preceding the measurements did not differ much among species. Species‐specific 3‐day daytime temperatures before the PHT measurements ranged 20.2°C–21.9°C across sites and 16.3°C–19.5°C, 22.1°C–24.0°C and 20.5°C–23.6°C at the HE, ME and LE sites, respectively.

Chlorophyll fluorescence measurements were made on detached, dark‐acclimated (for at least 30 min) leaves, using a portable Pocket‐PEA fluorimeter (Hansatech Instruments, King's Lynn, United Kingdom) with a light pulse intensity of 3500 μmol photons m^−2^ s^−1^ and duration of 1 s. The instrument records data every 10 µs during the measurement. The measurements were carried out inside huts at each field site on the same day of leaf sampling. Each leaf was initially dark acclimated for 30 min using leaf clips before being measured at a non‐stress ambient temperature (20°C–25°C). After the ambient temperature measurement, each leaf was again dark acclimated inside the humid plastic bags in the cooling box for 30 min. Due to logistical constraints, not all leaves could be measured simultaneously; thus, the interval between leaf sampling and measurement varied, with the total time span between the first and last measurement extending up to 8 h. However, previous work by Tarvainen et al. (2022) on two species (*Hmo* and *Sgu*, Table 1) found that neither *F*_0_ nor *F*_v_/*F*_m_ was significantly affected by the leaf detachment or the waiting time.

After the second dark acclimation, each leaf was taken from the plastic bags and was heated and measured at one target temperature. Heating was achieved using infra‐red lamps (IR/PAR red 175 W, Albert Kerbl GmbH, Buchbach, Germany). The leaves were placed horizontally on flexible arms a few centimetres from the metal shield covering the lamp (Figure S1). Leaf temperature was measured on the lower side of each leaf using a Testo 905i thermometer with a type K thermocouple (Testo, Lenzkirch, Germany). Leaf temperature was recorded every 2 s and increased gradually to prevent overshooting the target temperature. The infra‐red lamps were regulated by dimmers to control the heating process. The *F*_v_/*F*_m_ measurement was taken directly after (within a couple of seconds) the *T*_leaf_ had been within ±1°C of the target temperature, with a maximum amplitude variation of less than 1°C, for 2 min. Each measurement took between 4 and 20 min, depending on the target temperature. The rate of *T*_leaf_ increase was thus faster than the 1°C/min rate commonly used in studies with sequential heating in water baths (Krause et al. 2010). Additional details about the method can be found in Tarvainen et al. (2022).

### 2.3 Measurements of Recovery From Heat Stress

As described above, we measured chlorophyll fluorescence temperature responses immediately after the heat treatment, instead of measuring the longer‐term effects of heat stress after a 24‐h recovery period, which is the case in the commonly used protocol (Krause et al. 2010). To compare the immediate effect of heat stress and the effect remaining after a recovery period, we conducted two additional experiments. In the first one, we used seedlings of two species, *Mla* and *Paf* (Table 1), grown in 2‐l pots in climate chambers in Gothenburg, Sweden (Percival Scientific, CLF Plant Climatics). The seeds for this experiment were collected near the research plantations at the HE site in Rwanda. The plants were grown at a temperature of 20°C/15°C (day/night) on a 12‐h light/dark cycle. During the day, the plants were exposed to a light intensity of 400 μmol photons m^−2^ s^−1^. They were watered three times a week, and nutrient application was carried out on a weekly basis to ensure adequate water and nutrient availability. We sampled 7‐8 leaves from each of six plants per species, totalling 45 leaves per species. Five leaves were measured at nine target temperatures (38°C, 42°C, 44°C, 46°C, 48°C, 50°C, 52°C, 54°C and 56°C), as well as at ambient temperature (20°C). Measurements and heating treatment were conducted as described in the previous section, with the addition that *F*_v_/*F*_m_ was also measured at 20°C–25°C after a recovery period of 24 h at room temperature and darkness inside humid plastic bags.

In a second recovery experiment, sun‐exposed leaves of Rwanda TREE trees of all the species listed in Table 1 were measured for *F*_v_/*F*_m_ after a heat treatment corresponding to the site‐ and species‐specific *T*_50_ values determined in the main campaign (values in Figure S2). This was done in June 2022 for trees in the control plots at the ME site only, using the measurement and heating protocols described in the previous section, with the addition that *F*_v_/*F*_m_ was also measured at 20°‐25°C after a recovery period of 4, 8, 24 and 48 h at room temperature and darkness inside humid plastic bags.

### 2.4 Leaf Temperature Measurements

The leaf temperatures of sun‐exposed leaves were measured with infra‐red thermometers during three campaigns covering both wet and dry periods: February–March 2020 (wet season), June–September 2020 (dry season) and June–September 2021 (dry season). Measurements were made from 10:00 to 15:00 on sunny days (Photosynthetic photon flux density above 1000 µmol m^−2^ s^−1^), on mature, healthy, sun‐exposed leaves facing the sun at a perpendicular angle, or as close to that as possible. These measurements and data have been described by Manzi et al. (2024).

### 2.5 Leaf Morphology and Stomatal Conductance

Before chlorophyll fluorescence measurement, leaf length and width were measured on all harvested leaves using a ruler. Based on these data, leaf area was calculated using the species‐specific leaf area factors reported by Manishimwe et al. (2022). For LMA, we used species‐specific data from 2018 to 2019 reported in another Rwanda TREE study (Manishimwe et al. 2022).

Leaf *g*_s_ was measured on attached sun leaves of 5–6 plants per species and site during 9:00–15:00 h in February 2020, using a LI‐6400 portable photosynthetic system (Li‐Cor Biosciences, Lincoln, NE, United States). The measurements for all species and sites were done at a *T*_leaf_ of 25°C–30°C, a CO_2_ concentration of 415 ppm and saturating light of 1800 µmol m^−2^ s^−1^ inside the leaf chamber. The relative humidity was 50%–80%. To obtain *g*_s_ values representative for natural conditions, the gas exchange measurement was made within a couple of minutes after a leaf was inserted into the LI‐6400 leaf chamber, which is not sufficiently long for stomata to respond much to the chamber conditions. These measurements were described in more detail by Wittemann et al. (2024).

### 2.6 Thylakoid Membrane Lipid Composition and Osmolality

Leaf discs for membrane lipid and osmolality determination were sampled with a 10 mm puncher in November 2018. Three 10‐mm discs from one leaf per tree were sampled on five plants per species and site. The sample collection was carried out predawn and the sampled discs were immediately stored in aluminium foil envelopes placed in a liquid nitrogen dry shipper (−196°C). After transport to the lab in Sweden, the samples were stored at −80°C until extraction. The membrane lipids were extracted from leaf discs using a modified Bligh and Dyer extraction protocol and analysed by LC‐MS/MS as previously described (Wittemann et al. 2022). The absolute amounts of different lipid classes (monogalactosyldiacylglycerol, MGDG; digalactosyldiacylglycerol, DGDG; phosphatidylglycerol, PG; sulfoquinovosyl diacylglycerols, SQDG) were determined. Membrane lipid phase fluidity was evaluated based on the average number of double bonds (double bond index, DBI) in each lipid class. A decrease in DBI indicates a higher degree of saturation and thus increased membrane stability.

Osmolality was determined for leaf discs using a VAPRO 5600 vapour pressure osmometer (Wescor, Logan, UT, United States) following the protocol and equations provided by Bartlett et al. (2012) but with sampling of leaves predawn instead of applying a rehydration protocol on branches in the lab. Osmolality sampling and analyses were presented in more detail by Wittemann et al. (2024).

### 2.7 Data Analysis

All analyses were performed in R (R Core Team, 2020). To estimate each species’ *T*_crit_, *T*_50_ and *T*_95_, we followed the protocol from Perez et al. (2021). We fitted the relationship of *F*_v_/*F*_m_ versus leaf temperature for each species using Equation (1) and the ‘nls’ function in base R's ‘stats’ package. We generated bootstrapped means for *T*_crit_, *T*_50_ and *T*_95_, by randomly resampling data and fitting a new model for each species 1000 times. We present the mean bootstrapped values for *T*_crit_, *T*_50_ and *T*_95_ (Figure S2). More details on the method are described in Perez et al. (2021).

The TSM was determined by calculating the difference between species *T*_50_ and *T*_leafmax_. Each species’ *T*_leafmax_ was calculated as the mean of the values in the upper quartile of the leaf temperatures measured at each site (Manzi et al. 2024). In this study, full acclimation is defined as an equal magnitude of temperature change in *T*_50_ and *T*_leafmax_ for a given species along the temperature gradient.

Since our species are primarily categorised into two main successional groups, we first tested the effect of successional group (excluding the Mid‐successional group, which included only one species) using a mixed‐effects ANOVA on all traits (*T*_crit_, *T*_50_ and *T*_95_, lipids, *T*_leafmax_ and TSM). In this model, site and successional group were treated as fixed factors, and species was included as a random factor, and we used tree‐level data for each species and site. Since none of the analyses showed any significant effect of successional group, this factor was dropped from subsequent analyses and only the site factor was retained.

**Figure 1:**
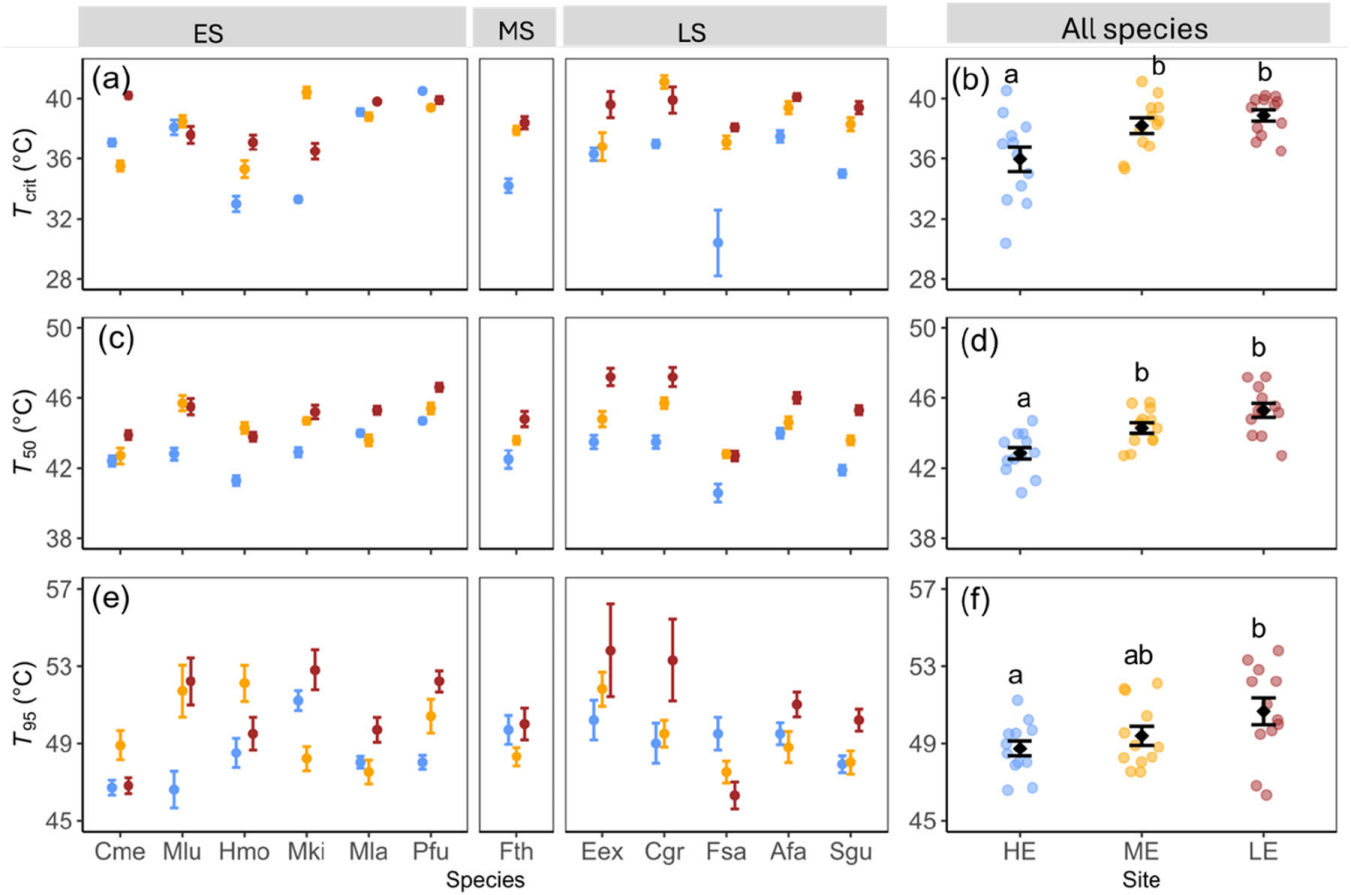
Heat tolerance thresholds of photosynthesis of 12 species (*n* = 5–6) grown at three sites in Rwanda TREE shown for each species separately (a, c, e) or across all species (b, d, f). Species are grouped as early successional (ES), mixed successional (MS) or late successional (LS). Data points in b, d, f represent the mean value for each species at each site. The (a, b) *T*_crit_ parameter represents the temperature at which the slope of the *F*_v_/*F*_m_ versus temperature relationship reached 15% of its steepest value, while (c, d) *T*_50_ and (e, f) *T*_95_ parameters indicate the temperatures that caused 50% or 95% reductions in *F*_v_/*F*_m_ compared to the control value, respectively. Different colours indicate different sites (blue: HE, high‐elevation site; yellow: ME, mid‐elevation site; red: LE, low‐elevation site). Species abbreviations are defined in Table 1. Different letters above data in b, d, f represent significant differences between sites across species (Tukey post hoc test, *p* < 0.05). Error bars represent standard errors of means.

**Figure 2:**
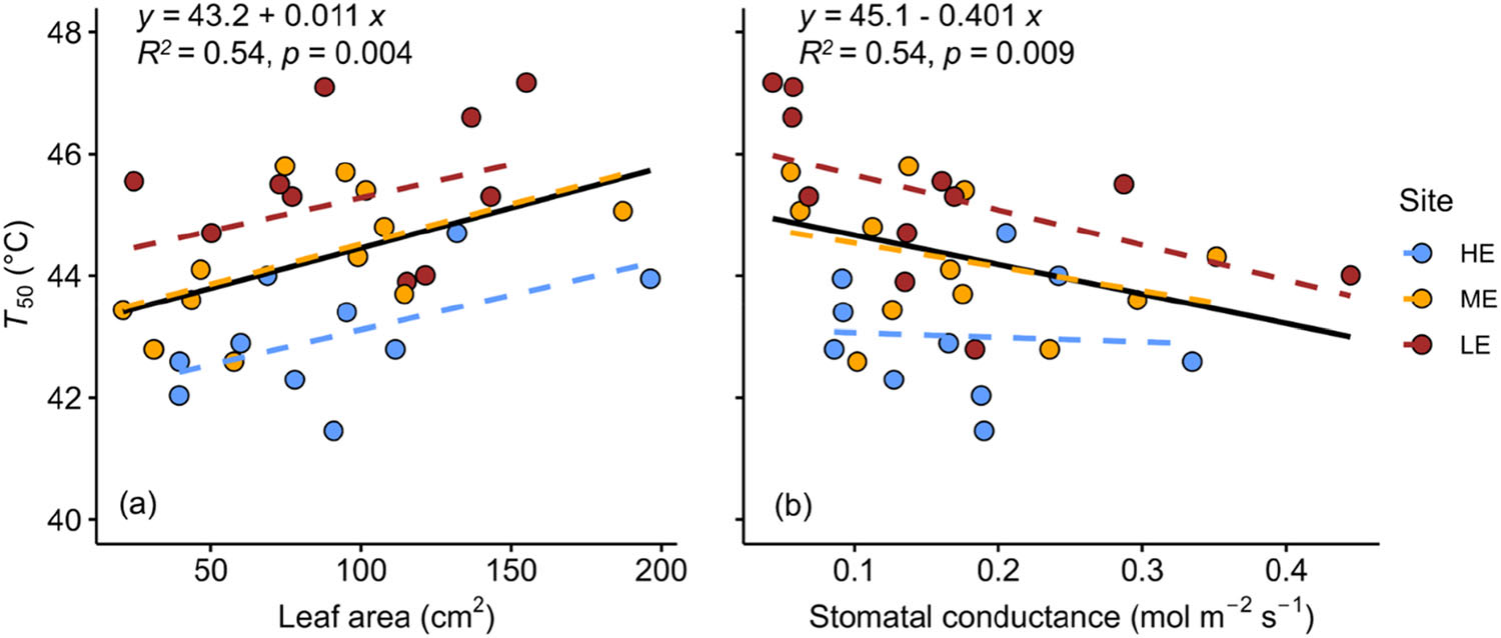
Dependencies of *T*_50_ on (a) leaf area and (b) stomatal conductance (*g*_s_). The equation, *R* ^2^ and *p* values provided are for the overall regression across sites (black solid line). Regression slopes did not significantly differ between sites. Data points represent the mean value for each species at each site. Dashed coloured lines denote site‐specific linear regressions. Blue symbols indicate HE, high elevation site; yellow: ME, mid‐elevation site and red: LE, low‐elevation site. [Color figure can be viewed at wileyonlinelibrary.com]

**Figure 3:**
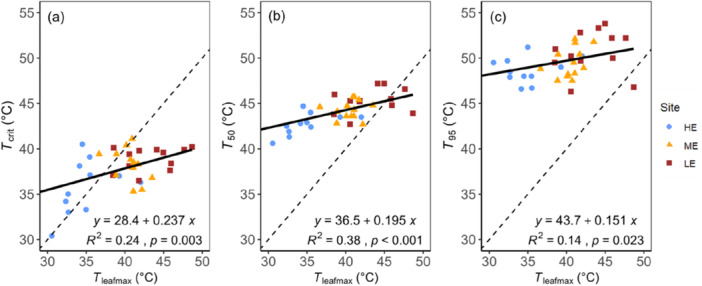
Variation in (a) *T*_crit_, (b) *T*_50_ and (c) *T*_95_ plotted against *T*_leafmax_. Values below the 1:1 line indicate exceedance of the given temperature tolerance threshold. Significant slopes of the regression lines fitted to the pooled data indicate responses of *T*_crit_, *T*_50_ and *T*_95_ to prevailing maximum leaf temperatures. Data points represent the mean value for each species at each site. Blue circles represent the high‐elevation site, yellow triangles indicate the mid‐elevation site, and red squares correspond to the low‐elevation site. [Color figure can be viewed at wileyonlinelibrary.com]

**Figure 4:**
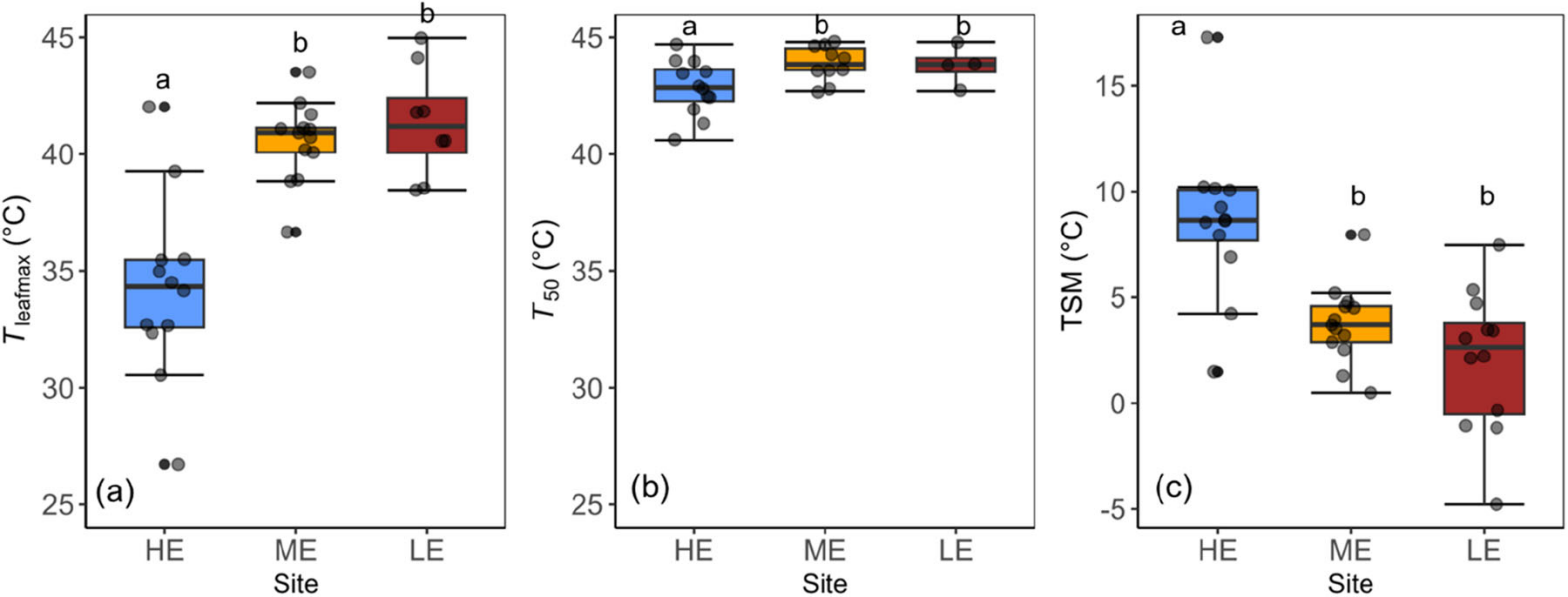
Between‐site variation in (a) maximum leaf temperature (*T*_leafmax,_°C), (b) leaf temperature at which the *F*_v_/*F*_m_ is reduced by 50% (*T*_50_,°C) and (c) thermal safety margin (TSM,°C). The median (central line of the box), the 75th percentile (upper line), the 25th percentile (lower line) and the 90th and the 10th percentiles (whiskers) are shown. Grey data points indicate the mean value for each species at each site. Black dots denote outliers. Blue bars indicate HE, high elevation site; yellow ME, mid‐elevation site and red: LE, low‐elevation site). Different letters above boxplots represent significant differences among sites (Tukey post hoc test, *p* < 0.05). Error bars represent standard errors of means. [Color figure can be viewed at wileyonlinelibrary.com]

**Figure 5:**
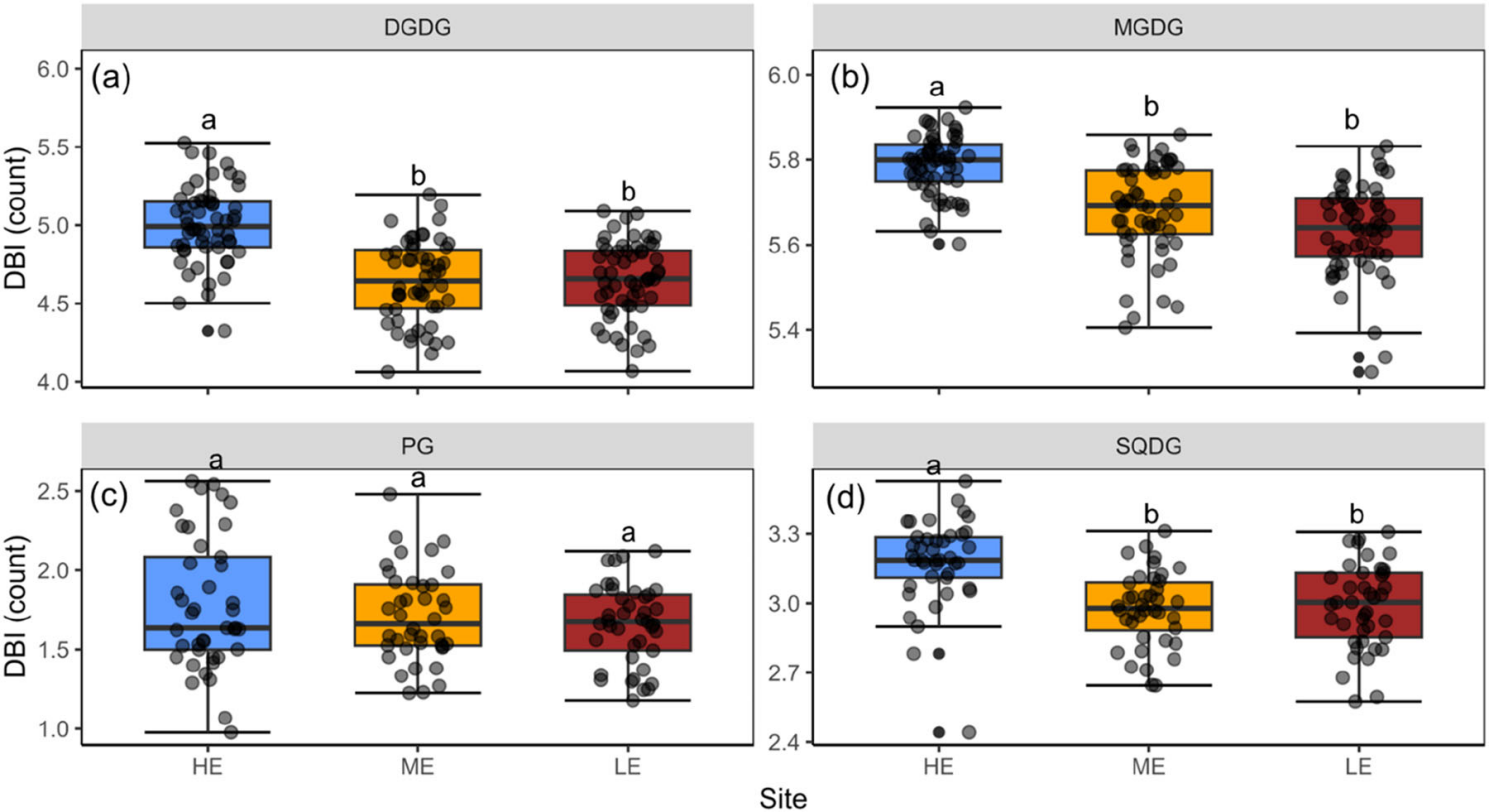
Between‐site variation in thylakoid membrane lipid composition with respect to the double bond index (DBI, count) for the lipid classes (a) digalactosyldiacylglycerol (DGDG), (b) monogalactosyldiacylglycerol (MGDG), (c) phosphatidylglycerol (PG) and (d) sulfoquinovosyl diacylglycerols (SQDG). Grey data points indicate the mean value for each species at each site. Black dots denote outliers. Different sites are indicated by blue: HE, high elevation site; yellow: ME, mid‐elevation site; red: LE, low‐elevation site. Different letters above boxplots represent significant differences between sites (Tukey post hoc test, *p* < 0.05). Error bars represent standard errors of means. [Color figure can be viewed at wileyonlinelibrary.com]

**Figure 6:**
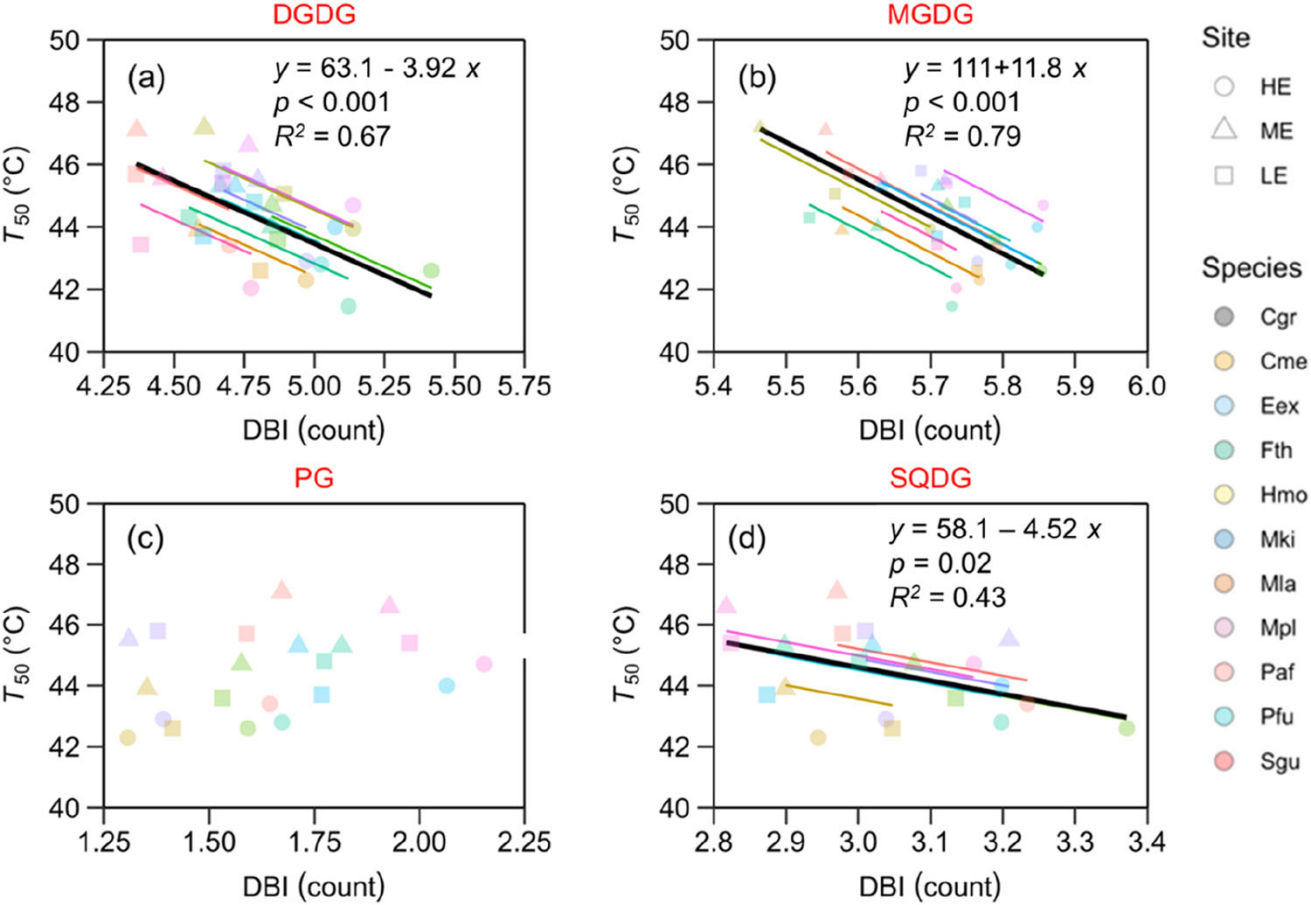
Variation in the temperatures at which the quantum yield of photosystem II (*F*_v_/*F*_m_) is reduced by 50% (*T*_50_,°C) plotted against the variation in thylakoid membrane lipid composition with respect to the double bond index (DBI, count) for the lipid classes (a) digalactosyldiacylglycerol (DGDG), (b) monogalactosyldiacylglycerol (MGDG), (c) phosphatidylglycerol (PG) and (d) sulfoquinovosyl diacylglycerols (SQDG). The equation, *R* ^2^ and *p* values provided are for the overall regression across species (black solid line). Regression slopes did not significantly differ between sites. Data points represent the mean value for each species at each site. Different shapes indicate different sites: circles for HE, high elevation site; triangles for ME, mid‐elevation site and squares for LE, low‐elevation site. Different colours indicate different species. [Color figure can be viewed at wileyonlinelibrary.com]

To test the effect of site on *T*_crit_, *T*_50_, *T*_95_, lipid content and TSM (Figures 1, 4 and 5), we used a one‐way ANOVA with site as the fixed effect, based on species means at each site, followed by Tukey's honest test for post hoc comparisons. We further assessed the relationships between *T*_50_ and leaf area and *g_s_* (Figure 2), as well as between *T*_leafmax_ and *T*_crit_, *T*_50_ and *T*_95_ (Figure 3), using simple linear regression models on species means at each site. Lastly, we tested the relationships between PHT parameters (*T*_crit_, *T*_50_ and *T*_95_) and different classes of membrane lipids (Figure 6), as well as leaf osmolarity, using mixed‐effects regression models using *nlme* R package (Pinheiro et al. 2023). In these models, PHT parameters were the response variables; lipid classes and leaf osmolality were included as fixed effects, and species was included as a random effect, using species means at each site. Only random intercepts were considered, as preliminary analyses indicated that including both slopes and intercepts overparameterized the model. Effects were considered statistically significant at *p* ≤ 0.05. For data import, we used *readxl* (Wickham and Jennifer 2023). To make plots, we used the following R packages: *ggplot2* (Wickham 2016), *Rmisc* (Hope 2013), *gridExtra* (Auguie and Antonov 2017) and *ggpubr* (Kassambara 2022).

## 3 Results

The heat tolerance thresholds *T*_crit_, *T*_50_ and *T*_95_ were generally significantly higher at the low‐elevation (LE) and medium‐elevation (ME) sites compared to the high‐elevation (HE) site for all species, except for *T*_crit_ of *Pfu* and *T*_95_ of *Fsa* (*p* < 0.05; Figure 1; Figure S2). The successional groups did not significantly differ in any of the PHT thresholds measured. Averaged across species, *T*_crit_ was significantly lower at the HE compared to ME and LE sites (*p* < 0.01), recorded as 35.6°C ± 0.82°C, 38.1°C ± 0.49°C and 38.9°C ± 0.37°C for HE, ME and LE sites, respectively (mean ± SE; Figure 1b). Within each site, *T*_crit_ differed among species and ranged between 30.4°C and 39.1°C, 35.3°C–41.1°C and 36.5°C–40.1°C for HE, ME and LE sites, respectively (Figure 1a). *T*_50_ differed between sites (*p* < 0.001, Figure 1c,d). On average across species, *T*_50_ was significantly lower at the HE site compared to the other sites, with values of 42.8°C ± 0.34°C, 44.3°C ± 0.27°C and 45.3°C ± 0.27°C for the HE, ME and LE sites, respectively. Furthermore, *T*_50_ showed interspecific variation within each site, ranging between 40.6°C and 44.7°C, 42.7°C–45.7°C and 42.7°C–47.2°C for the HE, ME and LE sites, respectively. *T*_95_ showed significant differences only between HE and LE sites (*F*_(2, 33)_ = 3.24, *p* = 0.04). *T*_95_ was 48.7°C ± 0.4°C, 49.4°C ± 0.46°C and 50.6°C ± 0.69°C and ranged between 46.6°C–51.2°C, 47.5°C–52.9°C and 46.3°C–53.8°C for HE, ME and LE sites, respectively (Figure 1e,f).

### 3.1 T_50_ and Leaf Thermoregulatory Traits (Stomatal Conductance and Leaf Size)

Next, we explored if the considerable interspecific variation in *T*_50_ was linked to key traits controlling the leaf energy balance (leaf size and *g*_s_) such that species with traits causing higher *T*_leaf_ will also show increased heat tolerance. There was a significant positive relationship between leaf area and *T*_50_ across species (*R* ^2^ = 0.54, *p* = 0.004) and a significant negative relationship between *g*_s_ and *T*_50_ (*R* ^2^ = 0.52, *p* = 0.009; Figure 2). The relationship between *T*_50_ and leaf area was stronger at the HE (*R* ^2^ = 0.37, *p* = 0.04) and ME sites (*R* ^2^ = 0.31, *p* = 0.06) than at the LE site (*R* ^2^ = 0.17, *p* = 0.21). On the other hand, the relationship between *g*_s_ and *T*_50_ was stronger at the LE site (*R* ^2^ = 0.30, *p* = 0.08) compared to the HE (*R* ^2^ = 0.01, *p* = 0.86) and ME sites (*R* ^2^ = 0.11, *p* = 0.31).

Regarding other PHT thresholds, *T*_95_ showed a significant relationship with leaf area but not with *g*_s_ and *T*_crit_ showed no correlation with either leaf area or *g*_s_ (Figure S3).

### 3.2 Acclimation of the PHT

The PHT thresholds *T*_crit_, *T*_50_ and *T*_95_ were positively related to *T*_leafmax_ regardless of site (Figure 3). At the ME and LE sites, *T*_crit_ equalled or was lower than the *T*_leafmax_ for all species except *Afa*. At the HE site, however, only three species (*Cgr*, *Eex* and *Mki*) surpassed their *T*_crit_. No species exceeded their *T*_50_ and *T*_95_ thresholds at the HE and ME sites. However, at the LE site, four species surpassed their T_50_ threshold, and one species (*Cme*) exceeded its *T*_95_ threshold (Figures 3 and S4).

Maximum *T*_leaf_ was 6.5°C–9.0°C higher at the lower‐elevation sites compared to the HE sites (Figure 4a) while the corresponding difference in *T*_50_ was only 1.4°C–2.4°C (Figure 4b). This indicates that *T*_50_ exhibited only partial acclimation, with an average increase of 0.36°C per 1°C rise in *T*_leafmax_ across species. Similarly, *T*_50_ increased by 0.31°C for each 1°C rise in growth temperature. Consequently, this led to narrower and sometimes negative TSM values at the lower‐elevation sites (Figure 4c). On average across species, TSM values based on *T*_leafmax_ data were 9.3°C, 4.6°C and 1.4°C for the HE, ME and LE sites, respectively. At the HE and ME sites, species displayed positive TSM values ranging from 1.5°C to 17.2°C and 0.5°C to 8.0°C, respectively (Figure 4). At the LE site, species exhibited both negative and positive TSM values ranging from −4.7°C to 7.5°C (Figure 4). If TSM was instead calculated using the maximum air temperature, it was on average 14.5°C, 12.6°C and 10.1°C at the HE, ME and LE sites, respectively (Figure S4).

A significant decrease in mean DBI across species was observed at the lower‐elevation sites for the thylakoid lipid classes DGDG, MDGD and SQDG (*p* < 0.001; Figures 5 and S5), but not for PG. In addition, significant site × species interactions were found for all lipid classes (*p* < 0.001; Figure S5), showing that the adjustment in lipid composition patterns to higher growth temperature varied among species. Furthermore, we observed a negative relationship between *T*_crit_, *T*_50_ and DBI for DGDG (*R* ^2^ = 0.48, *p* = 0.002; *R* ^2^ = 0.67, *p* < 0.001), MGDG (*R* ^2^ = 0.54, *p* = 0.009; *R* ^2^ = 0.79, *p* < 0.001) and SQDG (*R* ^2^ = 0.29, *p* = 0.02; *R* ^2^ = 0.43, *p* = 0.02, Figures 6 and S6). *T*_95_ was significantly only correlated with MGDG (*R* ^2^ = 0.365, *p* = 0.005) and slightly with DGDG (*R* ^2^ = 0.29, *p* = 0.09, Figure S7). In contrast, PG showed no significant relationship with any of the PHT thresholds (*R* ^2^ = 0.18, *p* = 0.08, *R* ^2^ = 0.14, *p* = 0.25, *R* ^2^ = 0.05, *p* = 0.806 for *T*_crit_, *T*_50_ and *T*_95,_ respectively; Figures 6, S6 and S7). No significant relationship was detected between osmolality and any of the PHT thresholds (*R* ^2^ = 0.03, *p* = 0.36; *R* ^2^ = 0.06, *p* = 0.23; *R* ^2^ = 0.11, *p* = 0.09 for *T*_crit_, *T*_50_ and *T*_95,_ respectively; Figure S8).

### 3.3 PHT and Safety Margins in Relation to LMA

**Figure 7:**
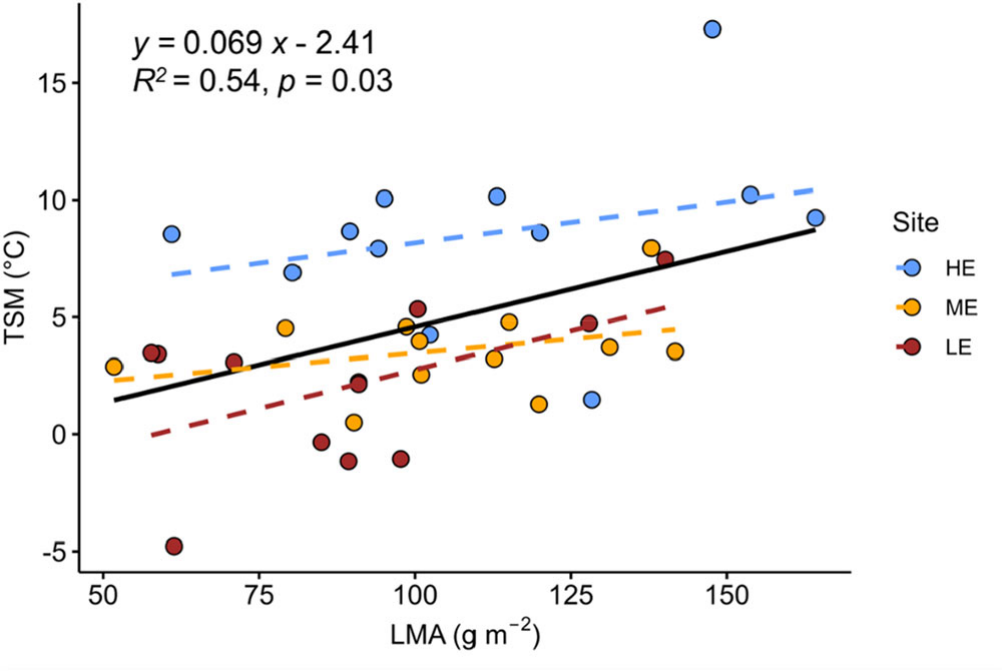
Relationship between leaf mass per area (LMA, g m^−2^) and thermal safety margin (TSM,°C). The equation, *R* ^2^ and *p* values provided are for the overall regression across sites (black solid line). Regression slopes did not significantly differ between sites. Data points represent the mean value for each species at each site. Dashed coloured lines denote site‐specific linear regressions. Blue symbols indicate HE, high elevation site; yellow: ME, mid‐elevation site and red: LE, low‐elevation site. [Color figure can be viewed at wileyonlinelibrary.com]

There was no statistically significant relationship between LMA and *T*_crit_, *T*_50_ and *T*_95_ (Figure S9). There was, however, a significant positive relationship between LMA and TSM, irrespective of site (*R* ^*2*^ = 0.54, *p* = 0.03, Figure 7).

### 3.4 Recovery From the Heat Stress

Our study revealed a significant difference in heat tolerance thresholds depending on if the measurement was performed 24 h after heat treatment compared to immediately following the heat stress (Figure S10). Compared to measurements directly after the heat pulse, the *T*_crit_ showed a 2.0°C increase when measured after 24 h in *Paf*, but no change for *Mla* (Figure S11). *T*_50_ was 2.7°C and 3.8°C higher when measured after 24 h compared to direct measurements for *Paf* and *Mla*, respectively. The *T*_50_ values were 43°C and 44.8°C for direct measurements, while they increased to 46.9°C and 47.5°C when measured after 24 h for *Mla* and *Paf*, respectively (Figure S10). Similar trends were observed for *T*_95_ in both species, with measurements taken after 24 h showing *T*_95_ values that were 9.3°C and 2.3°C higher for *Mla* and *Paf*, respectively, compared to direct measurements (Figure S11).

The recovery potential of *F*_v_/*F*_m_ was further evaluated using data from the measurement campaign at the ME site in which leaves had been exposed to a 2‐min heat pulse corresponding to their site‐ and species‐specific *T*_50_ values determined in the main campaign (values in Figure S2). Results showed that, on average across species, the direct *F*_v_/*F*_m_ decline was 40% while a 14% decline remained after 4 h (Figure S12).

## 4 Discussion

We examined the PHT of sun‐exposed leaves of 12 tropical tree species planted and grown along an elevation and temperature gradient in Rwanda. Our study revealed that species with traits causing higher leaf temperatures, such as large leaves and low *g*_s_, exhibited higher thresholds for direct photosynthetic impairment, but not high enough to prevent TSMs from narrowing in species with higher leaf temperatures (supporting Prediction 1). Moreover, we unveil that while the studied species possess the capacity to acclimate their PHT to warming, this acclimation falls short of mitigating the impact of rising leaf and growth temperatures. Species displayed narrower TSMs at the warmer sites, with four species even exhibiting negative values (supporting Prediction 2). Thylakoid membrane lipid saturation showed an important role in the acclimation of *T*_50_ to warming, with DBI being lower at warmer sites and negatively correlated with *T*_50_ (supporting Prediction 3). Moreover, we observed that TSM was influenced by leaf construction cost, such that species with higher LMA also had larger TSM (supporting Prediction 4).

### 4.1 Tree Species With Traits That Predispose Them to High Leaf Temperatures Have Higher PHT

Leaf size and *g*_s_ are important determinants of leaf temperatures, with leaves with higher leaf area and lower *g*_s_ warming up more than smaller leaves with higher *g*_s_ under sunny conditions because of reduced heat dissipation and evaporative cooling (Manzi et al. 2024). We predicted that species with traits conducive to higher *T*_leaf_ also exhibit enhanced thermotolerance. Indeed, we found that *T*_50_ increased with leaf size and decreased with *g*_s_ at all sites (Figure 2). This supports the idea that species with traits that predispose them for higher leaf temperatures also have higher temperature tolerance (Perez and Feeley 2020). This was further corroborated by a positive relationship between *T*_50_ and *T*_leafmax_ (Figure 3b), consistent with previous studies on neotropical species (Perez et al. 2021; Slot et al. 2021). It should be noted that although species with higher *T*_leaf_ had higher PHT, the former increased more than the latter resulting in decreased TSM in high‐*T*_leaf_ species, in line with the second part of our first prediction.

In this study, *T*_50_ values ranged from 40.6°C to 47.2°C, which is low compared to previously published results on tropical trees (Sastry and Barua 2017; Zhu et al. 2018; Feeley et al. 2020; Perez and Feeley 2020; Tiwari et al. 2021; Slot et al. 2021; Kullberg et al. 2024). The difference is at least partly due to differences in measurement protocols (Winter et al. 2025). While we measured chlorophyll fluorescence immediately after heat treatments to assess direct heat tolerance of photosynthesis, many prior studies followed the protocol developed by Krause et al. (2010), which evaluates irreversible damage to PSII by measuring heat effects after a 24 h recovery period. The recovery period of 24 h has been previously shown to increase *T*_50_ by approximately 2°C compared to direct measurements (Krause et al. 2010). Similar increases in *T*_50_ were found in this study; +2.7°C in *Mla* and +3.6°C in *Paf* (Figure S9). Much of the increase in *F*_v_/*F*_m_ during recovery occurs during the first 30 min after the heat pulse, probably reflecting that a large part of the direct effect is caused by instantaneous and reversible heat effects on the oxygen‐evolving complex (Tarvainen et al. 2022). While instantaneous heat response measurements of *F*_v_/*F*_m_ therefore do not determine thresholds for permanent PSII damage (Krause et al. 2010; Winter et al. 2025; Didion‐Gency et al. 2025), they offer valuable insight into the direct impact of heat stress on photosynthetic functioning. Such effects may be especially important in tropical field conditions, where leaves often endure prolonged daytime periods with high irradiance and temperature. To gain a deeper understanding of how heat stress affects plants, it is essential to consider both the short‐term and long‐term impacts on PSII. This can be achieved by routinely conducting measurements both directly after the heat exposure and after a 24 h recovery period in coming heat tolerance studies. Another possible reason for the relatively lower values of PHT thresholds reported in this study might be that growth temperatures at our sites are lower compared to other studies conducted in lowland tropical forests (Slot et al. 2021).

### 4.2 Partial Acclimation of PHT Leads to Narrower TSM

Despite increases in PHT thresholds (i.e., *T*_crit_, *T*_50_ and *T*_95_) at warmer sites, these were not enough to fully counteract the increase in growth‐ and leaf temperatures. For instance, while growth temperature and *T*_leaf_ increased by over 7°C, PHT thresholds only increased by 3.3°C, 2.5°C and 1.9°C for *T*_crit_, *T*_50_ and *T*_95_, respectively (Figure 3). Thus, for every 1°C rise in growth temperature, *T*_50_ increased by an average of 0.31°C, implying partial acclimation. This result is similar to findings from other studies, including a 0.38°C increase in *T*_50_ per 1°C rise in the warmest month's mean temperature in a global study (O'sullivan et al. 2017), a 0.34°C increase in *T*_crit_ per 1°C rise in growth temperature across 62 Australian species (Zhu et al. 2018), and a corresponding value of 0.34°C°C^−1^ across 147 species in Panama (Slot et al. 2021). However, one of these studies was conducted on plants grown in controlled‐environment chambers (Zhu et al. 2018) and the other two studies along natural temperature gradients could not separate between the influence of acclimation and adaptation. The present study is better suited to draw robust conclusions regarding thermal acclimation under field conditions as it is based on trees originating from the same plant material and grown freely rooted under field conditions at common gardens established along an elevation gradient. The similar shift in *T*_50_ per change in growth temperature in our study compared to previous studies which combine effects of both acclimation and adaptation (O'sullivan et al. 2017; Slot et al. 2021) implies either that our species have large acclimation capacity, or that shifts in previous temperature gradient studies are dominated by acclimation rather than adaptation. The latter was found to be the case in a global meta‐analysis on optimal temperatures of photosynthesis (Kumarathunge et al. 2019). Our finding of narrower TSMs at the warmer sites supports the idea that tropical trees are operating closer to their photosynthetic heat limits in a warming climate (Krause et al. 2010; Perez and Feeley 2020). This suggests that tropical trees, which have been found to have relatively narrow TSMs compared with temperate and boreal species, will be at a greater risk of thermal stress under global warming compared to species from other biomes (Kitudom et al. 2022; Kullberg and Feeley 2022).

We found that TSMs based on air temperatures were much higher than those based on *T*_leaf_ data (Figure S3). Moreover, we recently showed in another study that canopy temperature data also strongly overestimates the TSMs of sun‐exposed leaves (Manzi et al. 2024). These findings reinforce the importance of using *T*_leaf_ when determining plant TSMs, as leaf temperatures can deviate significantly from air temperatures, sometimes by up to 15°C–20°C in sun‐facing leaves under still conditions (Fauset et al. 2018; Manzi et al. 2024).

Acclimation is crucial for tropical forest trees to cope with warming, impacting their carbon uptake and overall performance amidst global climate change (Feeley et al. 2023). Predictions suggest that effective acclimation could limit Amazonian tree diversity loss to under 30% despite severe warming and deforestation. Conversely, inadequate acclimation may result in diversity losses exceeding 75%, even under optimistic climate and land‐use change scenarios (Feeley et al. 2011, 2023). These projections often overlook intermediate scenarios, such as partial acclimation, which are frequently observed in natural settings (Way and Yamori 2014; Reich et al. 2016; Wittemann et al. 2022). Observations from diverse tropical ecosystems, including our study in Rwanda, increasingly support the prevalence of partial acclimation in both the optimum temperature of photosynthesis and thermal tolerance (Slot and Winter 2017; Kumarathunge et al. 2019; Dusenge et al. 2021; Tarvainen et al. 2022; Wittemann et al. 2022; Mujawamariya et al. 2023; Dusenge et al. 2025).

Incorporating partial heat tolerance acclimation into vegetation models would thus improve the realism and accuracy of forecasts, especially for tropical forests, which are both highly biodiverse and highly vulnerable to climate warming. Integrating a best‐available estimate of ~0.3°C increase in heat tolerance per 1°C of warming, consistent with the range observed in empirical studies (O'sullivan et al. 2017; Zhu et al. 2018; Tarvainen et al. 2022; Middleby et al. 2024) would offer a more nuanced alternative to binary assumptions of no or complete acclimation. However, our results also highlight considerable interspecific and site‐level variation in acclimation capacity. Therefore, while fixed empirical values can serve as a useful first approximation, especially for large‐scale modelling, flexible model parameterisations that allow acclimation strength to vary by climate zone, plant functional type, species group, or species may provide a more accurate representation.

### 4.3 Trees Acclimate to Growth Temperature by Adjusting the Thylakoid Membrane Fluidity

Previous studies have identified various biochemical and physiological mechanisms underpinning the acclimation of plant PHT (Hüve et al. 2006; Mathur et al. 2014; Zhu et al. 2018, 2024; Taylor et al. 2019), but the importance of these mechanisms for heat tolerance in the field remains uncertain. In this study, we observed a significant decrease in number of double bonds (DBI) in thylakoid membrane lipids at the warmer sites (Figures 5 and S5). Moreover, we observed a negative relationship between *T*_50_ and the DBI of different lipid classes, suggesting the thermal acclimation of *T*_50_ is linked to shifts in the fatty acid composition of thylakoid membrane lipids (Figure 6). This aligns with previous observations of a positive relationship between PHT and the abundance of saturated fatty acids, enhancing membrane stability and alleviating heat stress (Zhu et al. 2018, 2024). It is thus highly likely that lipid adjustments play a key role in the acclimation of tropical trees to warming, adding to possible further contributions by mechanisms not explored in this study, such as the production of volatile organic compounds, the induction of heat shock proteins and the accumulation of antioxidant enzymes (Teskey et al. 2015; Aspinwall et al. 2019; Pollastri et al. 2019; Taylor et al. 2019; Rodrigues et al. 2020; Zhu et al. 2024). To fully understand this complexity, future studies should explore a broader range of mechanisms and consider the unique ways in which different tree species acclimate their PHT to warming.

### 4.4 TSM Is Linked to Leaf Construction Costs

We predicted that TSM would be positively associated with LMA, a trait commonly used as a proxy for leaf construction costs and structural investments, and this prediction was supported by our results. This finding aligns with the theoretical framework of carbon economics, which posits that leaves with greater construction costs must operate safely within thermal limits to ensure sufficient return on investment before senescence or heat‐induced damage (Bison and Michaletz 2024). However, we did not find a positive relationship between LMA and PHT, as reported in some earlier studies (Godoy et al. 2011; Sastry and Barua 2017; Sastry et al. 2018; Slot et al. 2021; Li et al. 2022; H. Zhang et al. 2024), but not in all (O'sullivan et al. 2017; Fadrique et al. 2022; Münchinger et al. 2023; González et al. 2025). This suggests that while LMA may not always confer higher absolute PHT, it is linked to heat resilience through leaf energy balance mechanisms acting to widen the TSM. Since TSM captures the combined effects of both *T*_leaf_ and physiological thresholds, it offers a more integrative measure of leaf thermal risk. We therefore propose that, from a carbon economics perspective, selection may favour coordination between LMA and TSM, rather than between LMA and PHT, as a strategy to maximise carbon gain under warming conditions.

## 5 Conclusions

We took advantage of a unique elevation gradient experiment in the Afrotropics to examine the acclimation of the direct PHT in woody tropical tree species exposed to increasing growth temperatures. We found that species having leaves with traits that predispose them to being warmer (larger size and lower *g*_s_) also tend to be more tolerant to those high temperatures. Across sites, trees showed some ability to adjust their PHT to warming by altering the thylakoid membrane lipid saturation. However, this acclimation was partial and not enough to compensate for the increased air and leaf temperatures at warmer sites, leading to a narrowing of TSMs. Our study also demonstrated a positive relationship between LMA and TSM, indicating that leaves requiring higher structural investment are generally more heat‐tolerant than those representing a lower investment. In summary, the results of this study suggest that limited acclimation capacity will lead to narrow photosynthetic TSMs of tropical trees in a warming climate.

## Conflicts of Interest

The authors declare no conflicts of interest.

## Supporting information


**Figure S1:** Image of the heating set‐up used during the field measurements.
**Figure S2:** The quantum yield of PSII (*F*_v_/*F*_m_) as a function of leaf temperature for the studied species.
**Figure S3:** Dependencies of *T*_crit_ and *T*_95_ on leaf area and stomatal conductance (*g*_s_).
**Figure S4:** Thermal safety margins of the studied species based on different PHT thresholds with maximum leaf and air temperatures.
**Figure S5:** Among‐sites and ‐species variation in thylakoid membrane lipid composition.
**Figure S6:** Variation in the temperature at which the slope of the *F*_v_/*F*_m_ versus temperature relationship reached 15% of its steepest value (*T*_crit_) plotted against the variation in thylakoid membrane lipid composition.
**Figure S7:** Variation in the temperatures at which the quantum yield of photosystem II (*F*_v_/*F*_m_) is reduced by 95% (*T*_95_,°C) plotted against the variation in thylakoid membrane lipid composition.
**Figure S8:** Variation in the temperatures at which the quantum yield of photosystem II (*F*_v_/*F*_m_) is reduced by 50% (*T*_*50*_, °C) plotted against the variation in leaf osmolality (mmol kg ^−1^).
**Figure S9:** Relationship between leaf mass per area (LMA) and the heat tolerance thresholds. **Figure S10:** Effects of a 2‐min heat pulse on the quantum yield of photosystem II (*F*_v_/*F*_m_) in (a) *Maesa lanceolata* and (b) *Prunus africana* recorded directly after heat treatment (green), or 24 h after heat treatment (orange).
**Figure S11:** Variation in *T*_crit_, *T*_50_, and *T*_95_ measured directly and after 24 h recovery for *Mla* and *Paf*.
**Figure S12:** Recovery of dark‐adapted *F*_v_/*F*_m_ in leaves of studied species at the ME site over 48 h post‐heat exposure.

**Dataset S1.** The data used in the study.

## Data Availability

The data that supports the findings of this study are available in the supplementary material of this article (Dataset S1).
