# Supplementary material for: Photosynthetic Heat Tolerance Partially Acclimates to Growth Temperature in Tropical Montane Tree Species
Source: Plant Cell Environ. 2025 Jul 23;48(11):7848–61. doi: 10.1111/pce.70079 (PMC12502021; doi:10.1111/pce.70079)
Supplement: Supplementary file 1 — Figure S1: Image of the heating set‐up used during the field measurements. Figure S2: The quantum yield of PSII (F v/F m) as a function of leaf temperature for the studied species. Figure S3: Dependencies of T crit and T 95 on leaf area and stomatal conductance (g s). Figure S4: Thermal safety margins of the studied species based on different PHT thresholds with maximum leaf and air temperatures. Figure S5: Among‐sites and ‐species variation in thylakoid membrane lipid composition. Figure S6: Variation in the temperature at which the slope of the F v/F m versus temperature relationship reached 15% of its steepest value (T crit) plotted against the variation in thylakoid membrane lipid composition. Figure S7: Variation in the temperatures at which the quantum yield of photosystem II (F v/F m) is reduced by 95% (T 95,°C) plotted against the variation in thylakoid membrane lipid composition. Figure S8: Variation in the temperatures at which the quantum yield of photosystem II (F v/F m) is reduced by 50% (T 50 , °C) plotted against the variation in leaf osmolality (mmol kg −1). Figure S9: Relationship between leaf mass per area (LMA) and the heat tolerance thresholds. Figure S10: Effects of a 2‐min heat pulse on the quantum yield of photosystem II (F v/F m) in (a) Maesa lanceolata and (b) Prunus africana recorded directly after heat treatment (green), or 24 h after heat treatment (orange). Figure S11: Variation in T crit, T 50, and T 95 measured directly and after 24 h recovery for Mla and Paf. Figure S12: Recovery of dark‐adapted F v/F m in leaves of studied species at the ME site over 48 h post‐heat exposure. [file PCE-48-7848-s002.docx]

**Supplementary material**


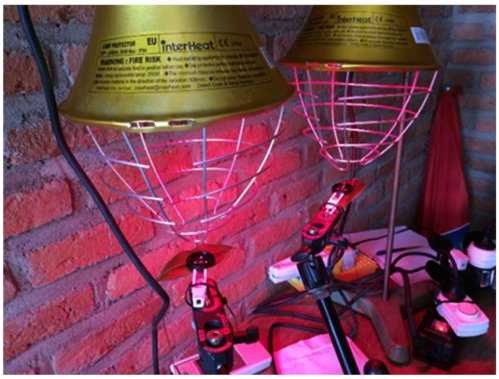


**Figure S1.** Image of the heating set-up used during the field measurements. Heating the leaves for thermal tolerance determination was achieved by infrared lamps. The heating effect was controlled by dimmers (lower right-hand corner of the photo) and by the distance between the leaf and the lamp. Leaf clips, equipped with thermocouples touching the abaxial side of the leaf, were used for dark adaptation before measurement with a Pocket PEA fluorimeter (Hansatech, King’s Lynn, U.K.). Photo: Lasse Tarvainen


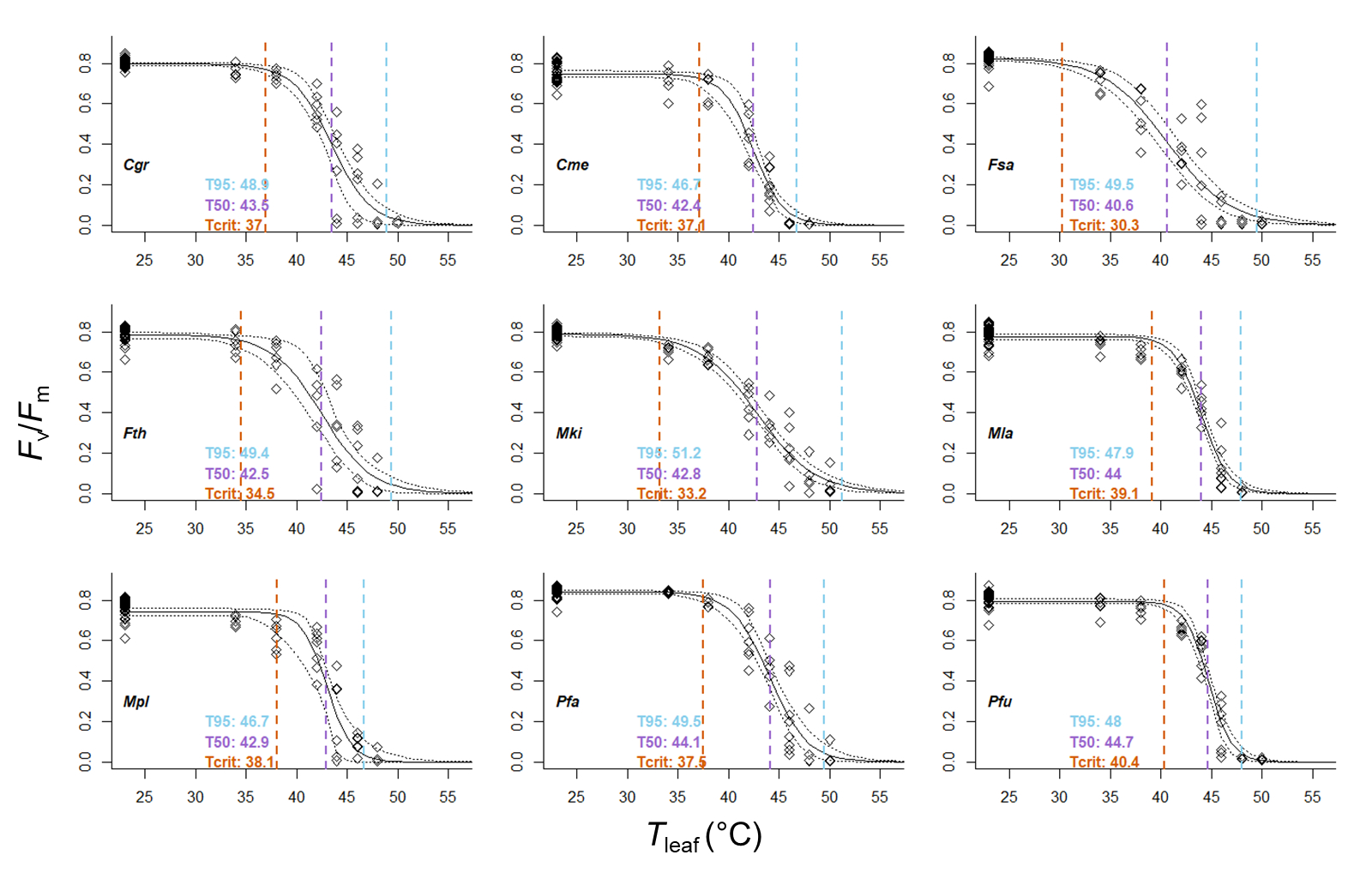


(a)


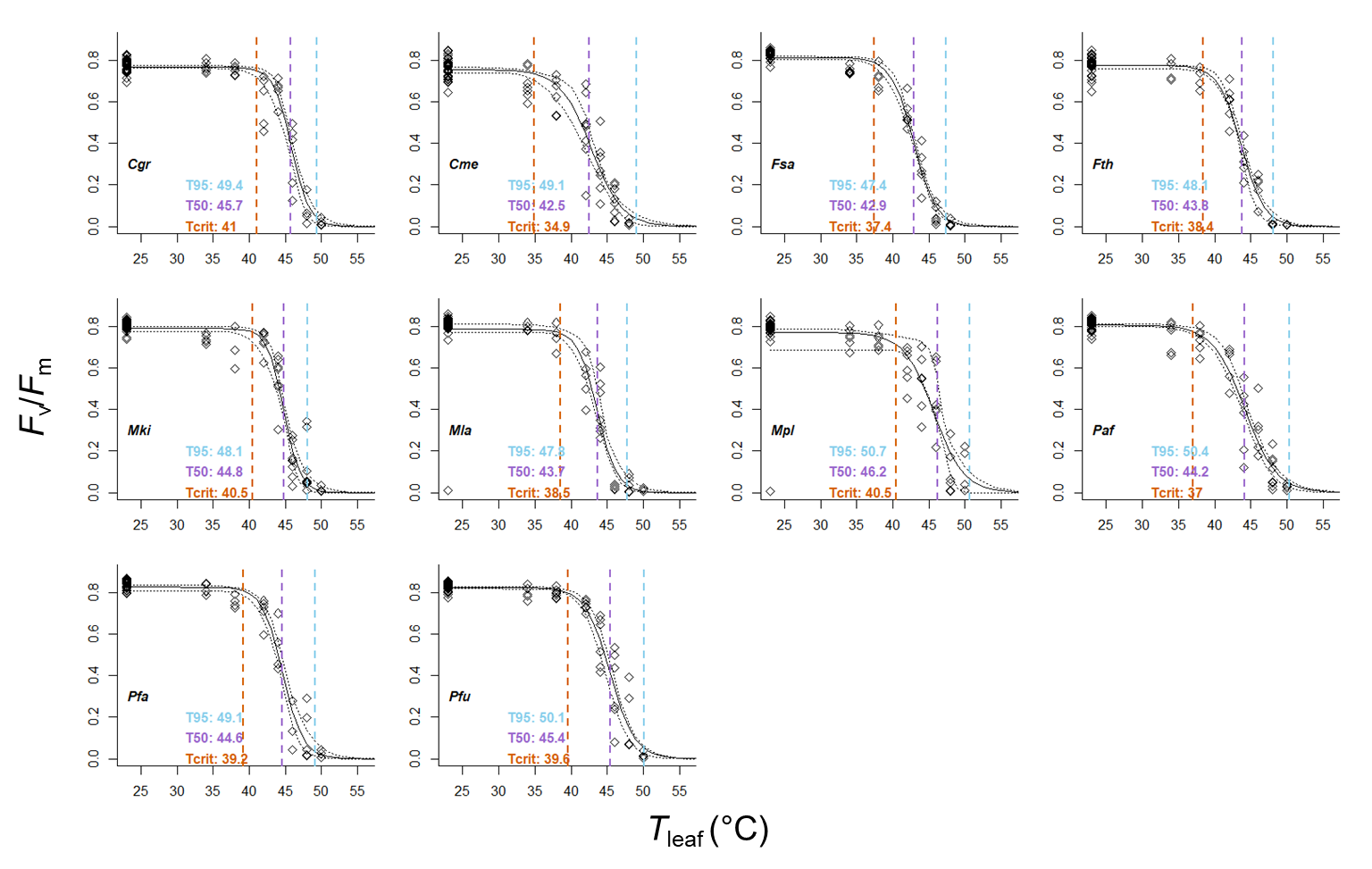


(b)

**
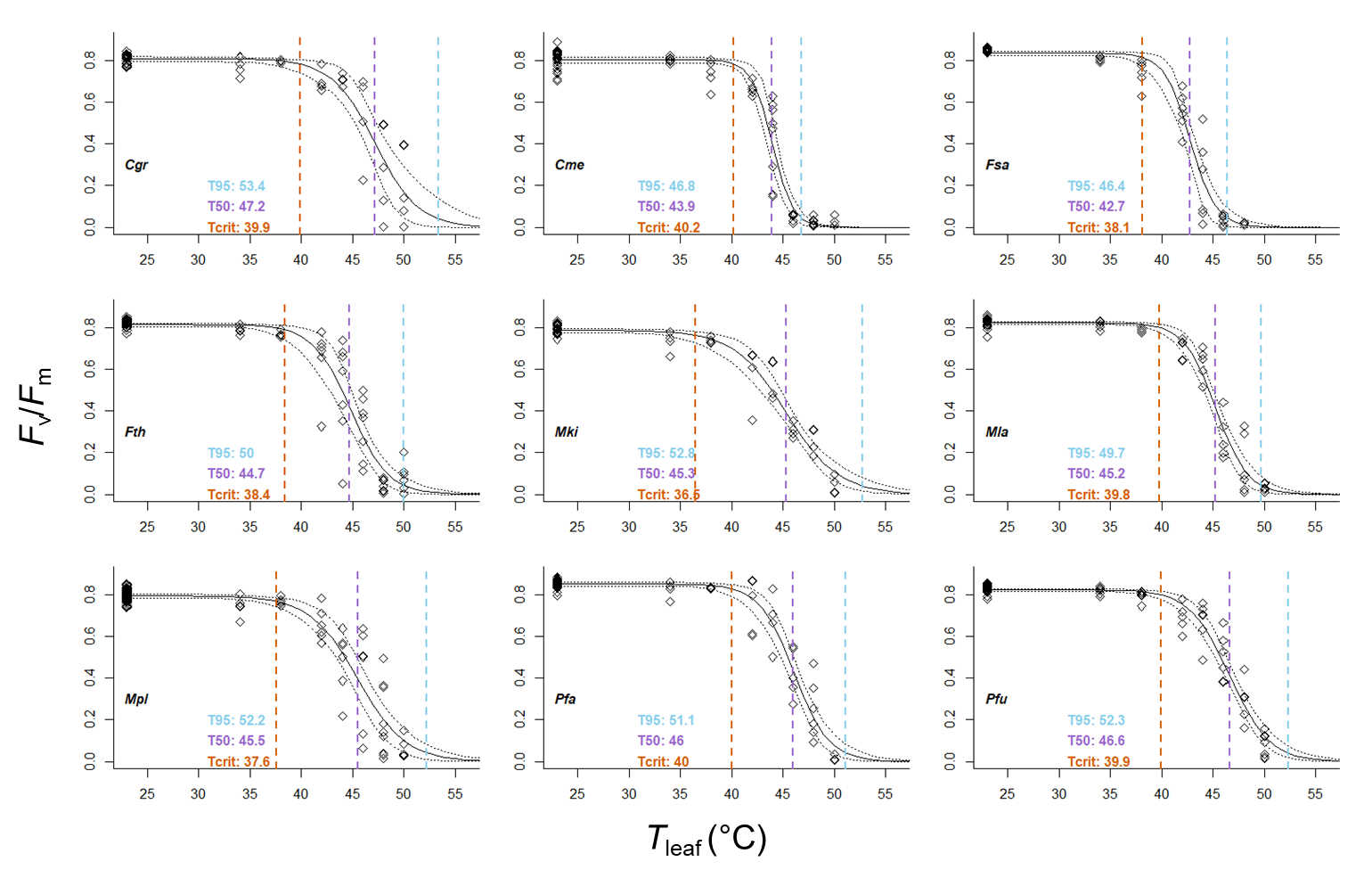
**

**(C)**

**Figure S2.** The dark-adapted quantum yield of photosystem II (F_v_/F_m_) as a function of leaf temperature, indicating the *T*_crit_, *T*_50_, and *T*_95_ heat tolerance thresholds for all species in the study at the (a) HE, (b) ME and (c) LE sites. Species abbreviations provided in the graphs are defined in Table 1.


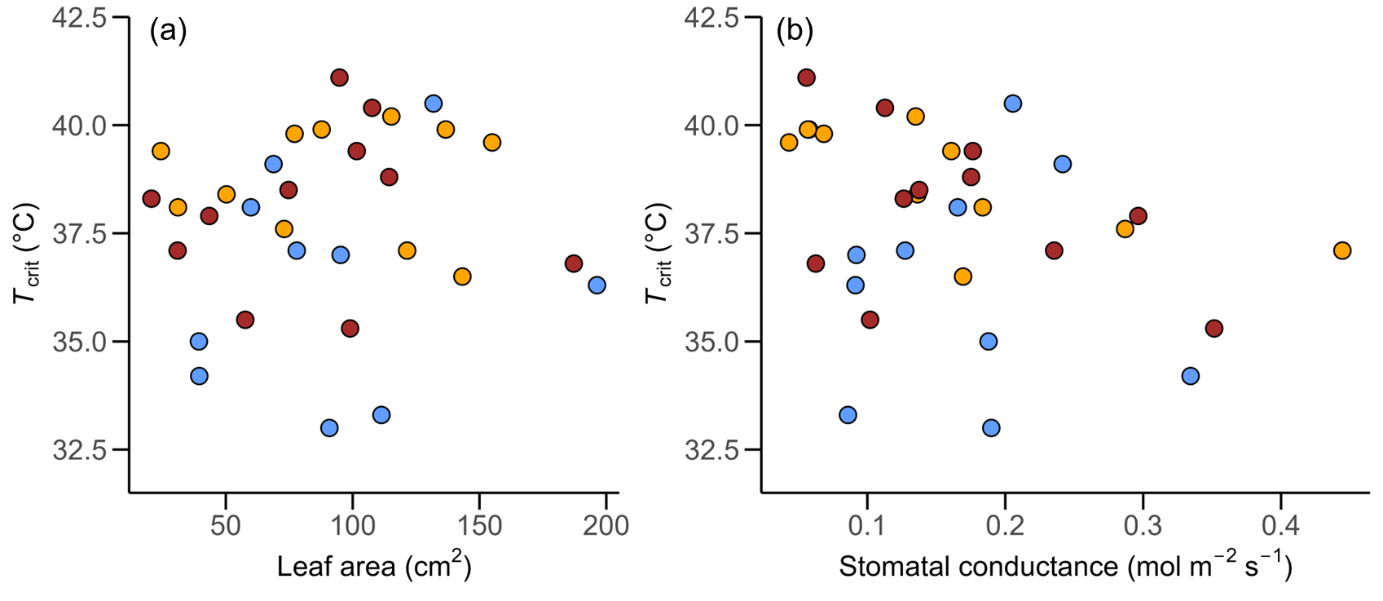


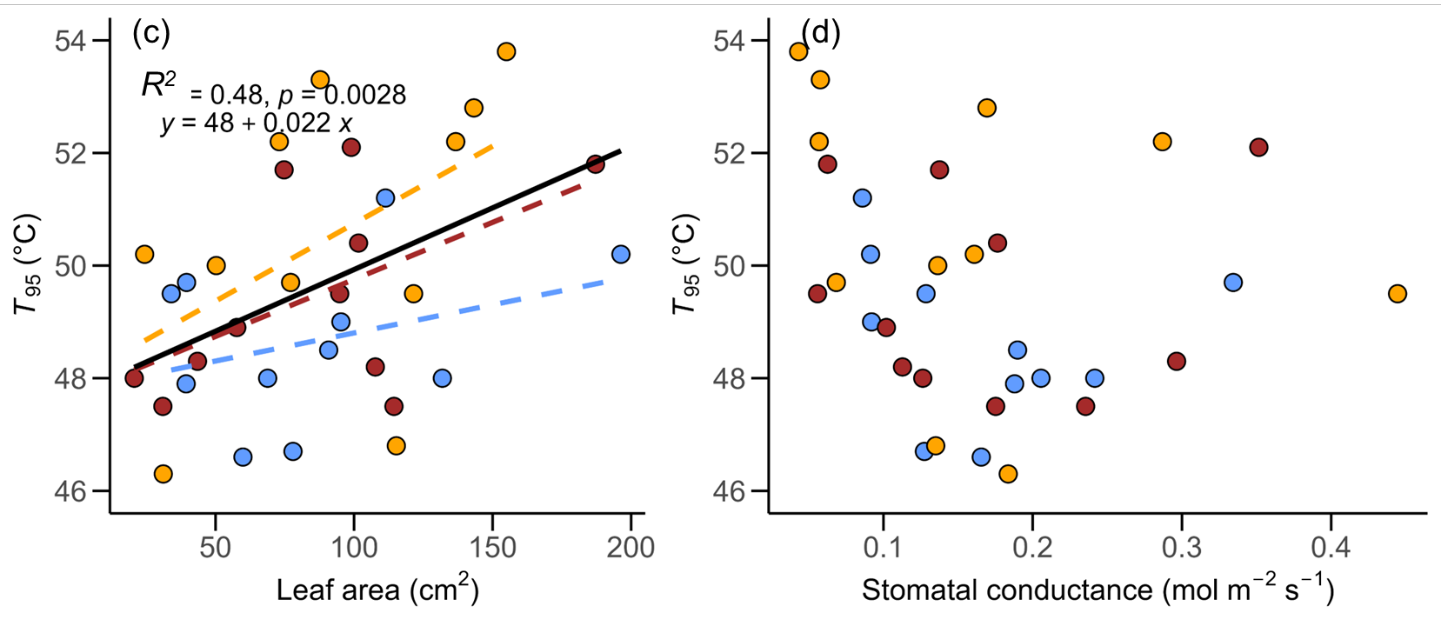


**Figure S3.** Dependencies of *T*_crit_ (a, b) and *T*_95_ (c, d) on leaf area and stomatal conductance (*g*_s_). The equation, *R*^2^ and *p* values provided are for the overall regression across sites (black solid line when significant). Regression slopes did not significantly differ between sites. Data points represent the mean value for each species at each site. Different colours indicate different sites (blue: HE, high elevation site; yellow: ME, mid-elevation site; red: LE, low-elevation site).


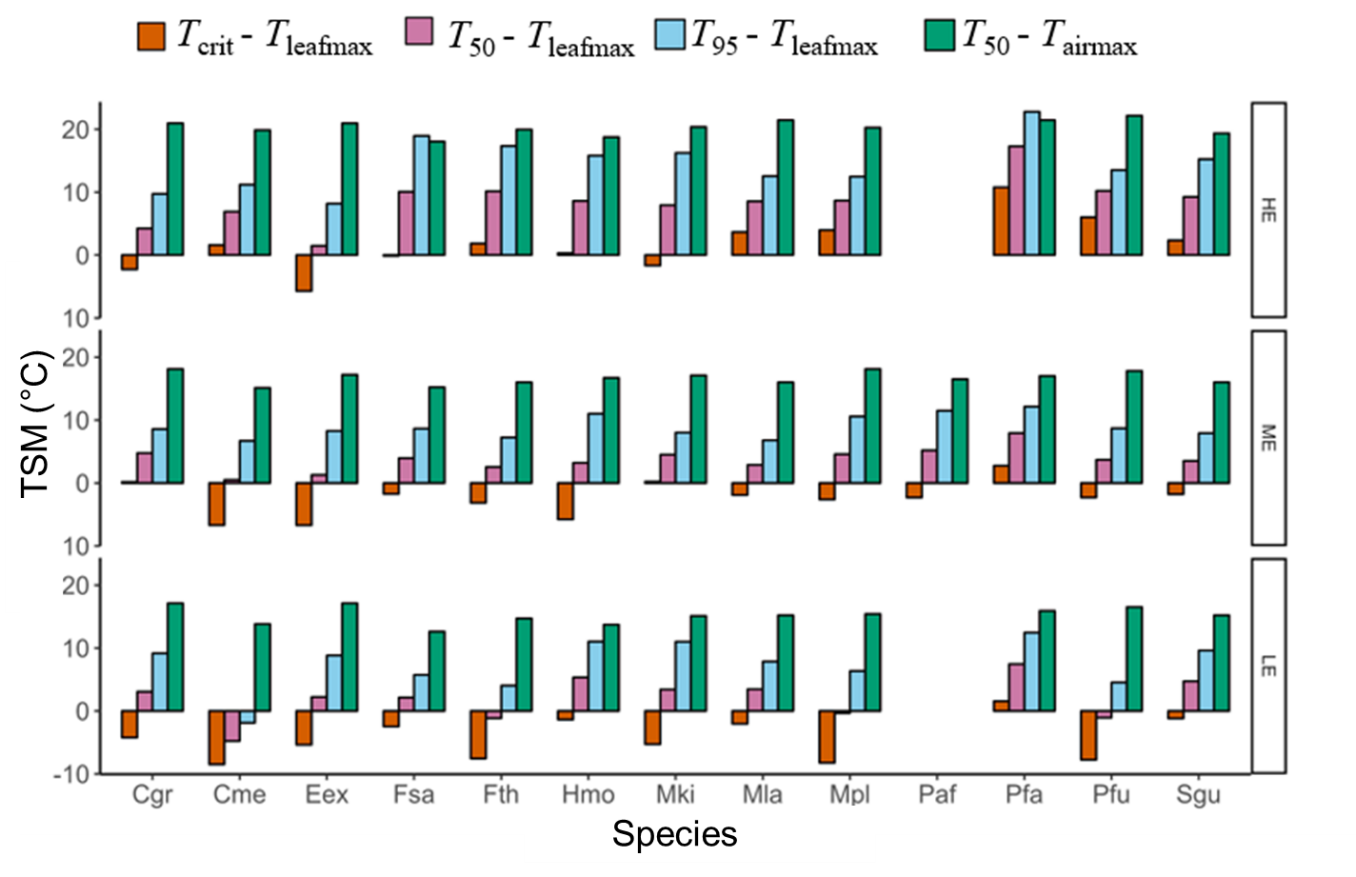


**Figure S4.** Thermal safety margins (TSM) for the studied species based on different photosynthetic heat tolerance thresholds and maximum leaf (*T*_leafmax_) and air temperatures (*T*_airmax_) at the high- (HE: on top), mid- (ME: in the middle) and low-elevation sites (LE: at the bottom). The *T*_leafmax_ represents the mean value of the highest quartile of observations made on sun-facing leaves in measurement campaigns described by Manzi *et al.* (2024), and *T*_airmax_ represents the mean value of the highest quartile of air temperatures measured at the same time in these campaigns. Different colors indicate different metrics used: orange: *T*_crit_ - *T*_leafmax_, pink: *T*_50_ - *T*_leafmax_, sky blue: *T*_95_ - *T*_leafmax_ and green: *T*_50_ - *T*_airmax_. Species abbreviations are defined in Table 1.


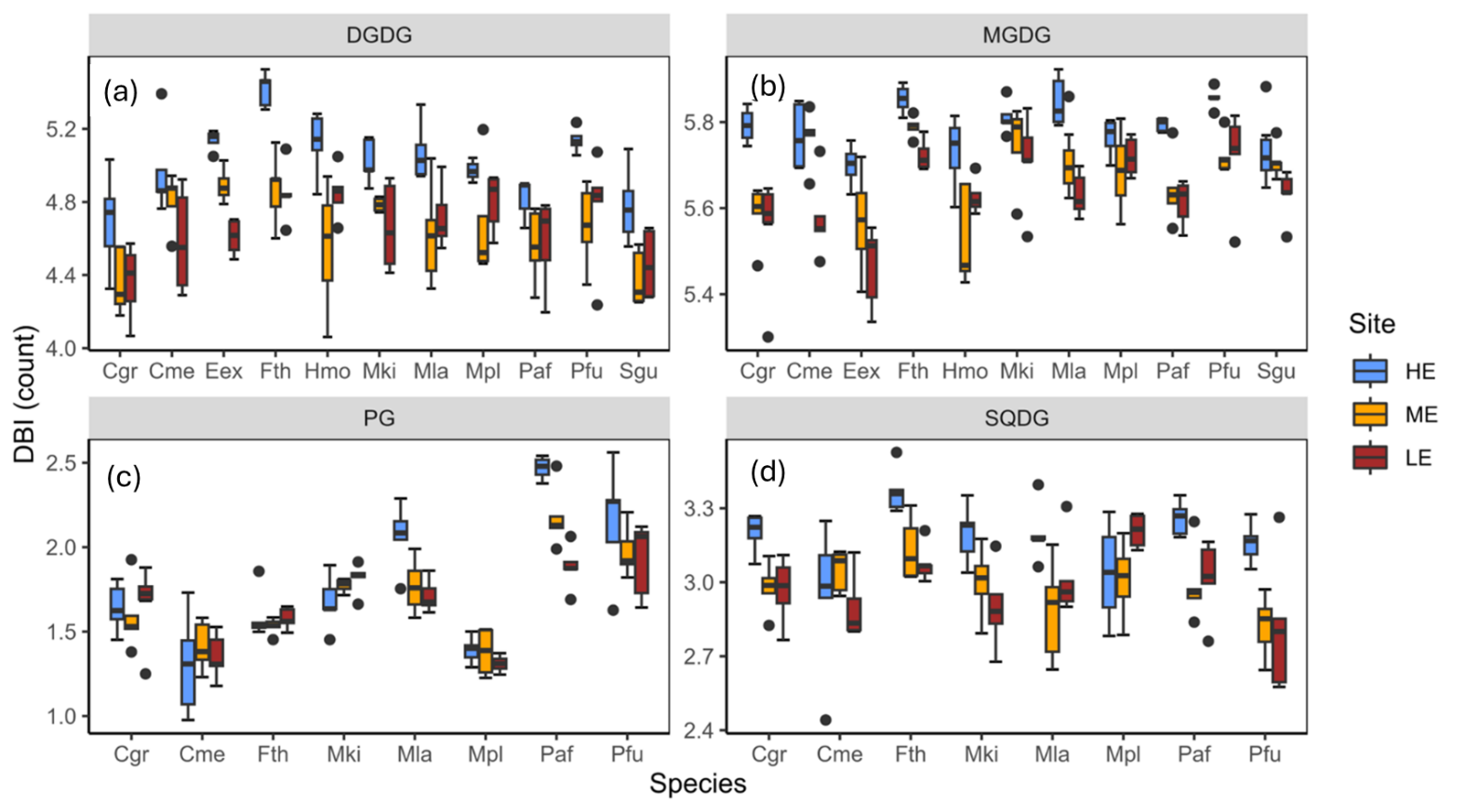


**Figure S5.** Among-site and -species variation in thylakoid membrane lipid composition with respect to the double bond index (DBI, count) for the lipid classes (a) digalactosyldiacylglycerol (DGDG), (b) monogalactosyldiacylglycerol (MGDG), (c) phosphatidylglycerol (PG) and (d) sulfoquinovosyl diacylglycerols (SQDG).  Different colours indicate different sites (blue: HE, high elevation site; yellow: ME, mid-elevation site; red: LE, low-elevation site). Species abbreviations are defined in Table 1. Error bars represent standard errors of means.


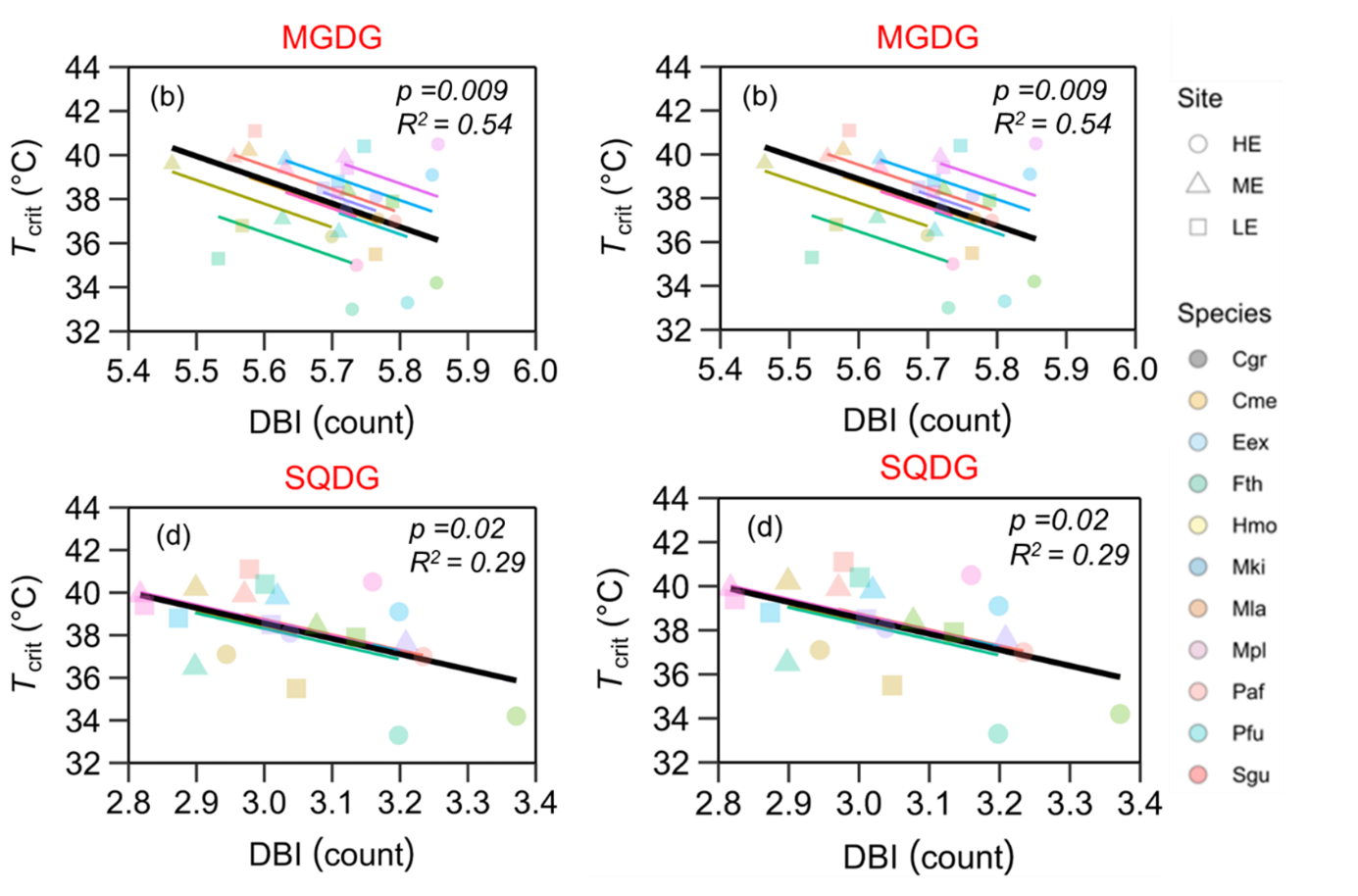


**Figure S6.** Variation in the temperature at which the slope of the *F*_v_/*F*_m_ versus temperature relationship reached 15% of its steepest value plotted against the variation in thylakoid membrane lipid composition with respect to the double bond index (DBI, count) for the lipid classes (a) digalactosyldiacylglycerol (DGDG), (b) monogalactosyldiacylglycerol (MGDG), (c) phosphatidylglycerol (PG), and (d) sulfoquinovosyl diacylglycerols (SQDG). The equation, *R*^2^ and *p* values provided are for the overall regression across species (black solid line). Data points represent the mean value for each species at each site. Different shapes indicate different sites: circles for HE, high elevation site; triangles for ME, mid-elevation site and squares for LE, low-elevation site. Different colours indicate different species. The solid black line indicates the overall regression.


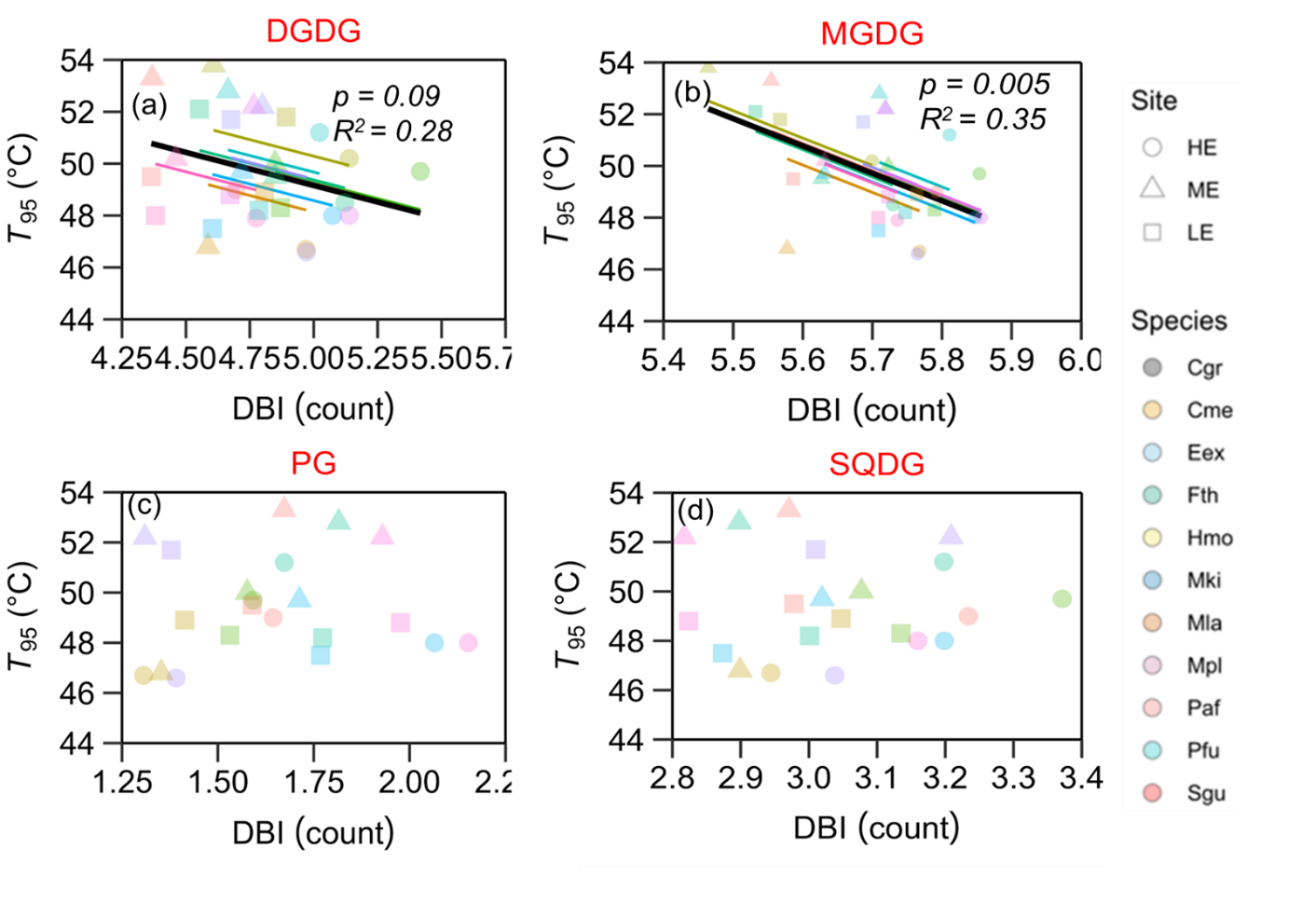


**Figure S7.** Variation in the temperatures at which the quantum yield of photosystem II (*F*_v_/*F*_m_) is reduced by 95% (*T*_95_, °C) plotted against the variation in thylakoid membrane lipid composition with respect to the double bond index (DBI, count) for the lipid classes (a) digalactosyldiacylglycerol (DGDG), (b) monogalactosyldiacylglycerol (MGDG), (c) phosphatidylglycerol (PG), and (d) sulfoquinovosyl diacylglycerols (SQDG). The equation, *R*^2^ and *p* values provided are for the overall regression across species (black solid line. Data points represent the mean value for each species at each site. Different shapes indicate different sites: circles for HE, high elevation site; triangles for ME, mid-elevation site and squares for LE, low-elevation site. Different colours indicate different species. The solid black line indicates the overall regression.


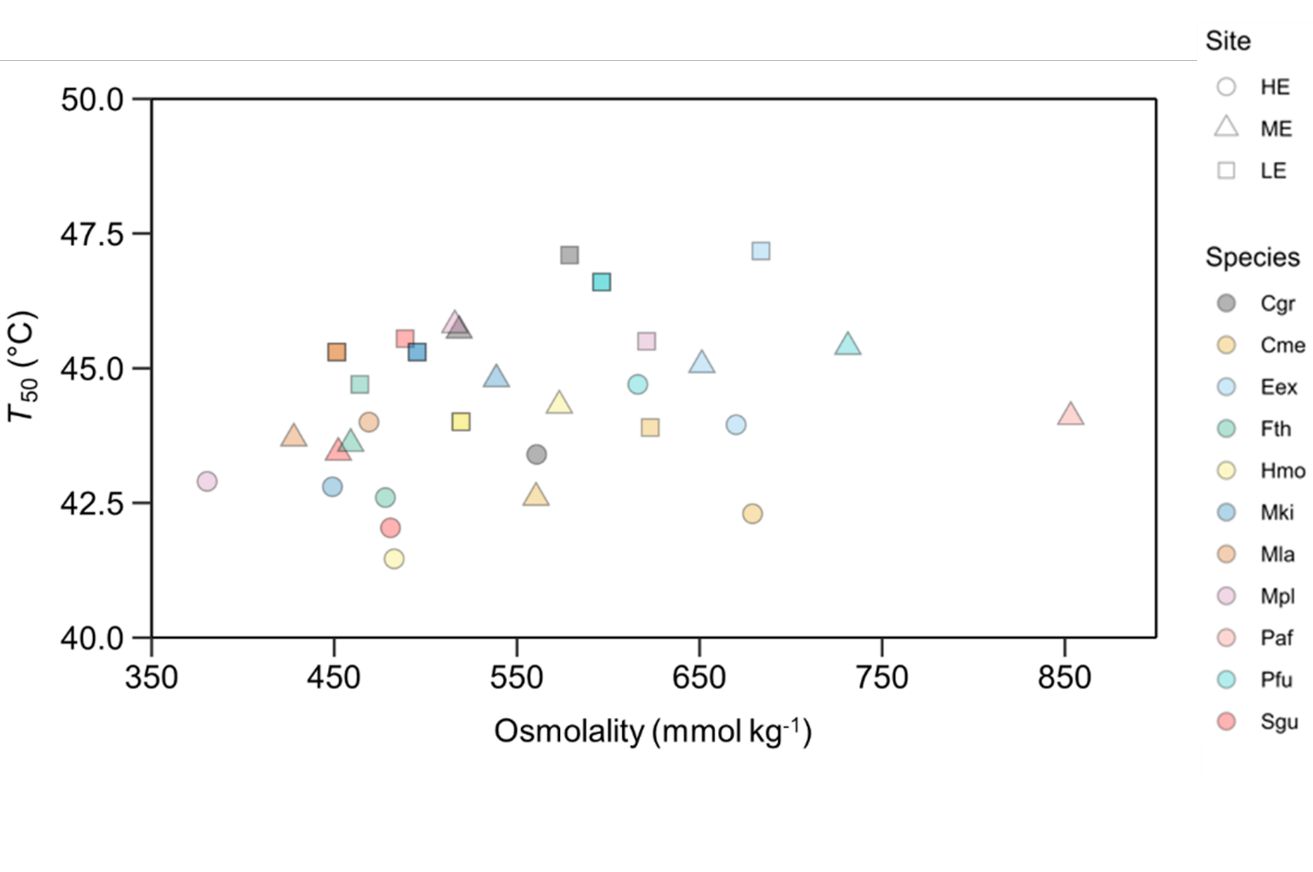


**Figure S8.** Variation in the temperatures at which the quantum yield of photosystem II (*F*_v_/*F*_m_) is reduced by 50% (*T_50_*, °C) plotted against the variation in leaf osmolality (mmol kg ^−1^). Data points represent the mean value for each species at each site. Different shapes indicate different sites: circles for HE, high elevation site; triangles for ME, mid-elevation site and squares for LE, low-elevation site. Different colours indicate different species.


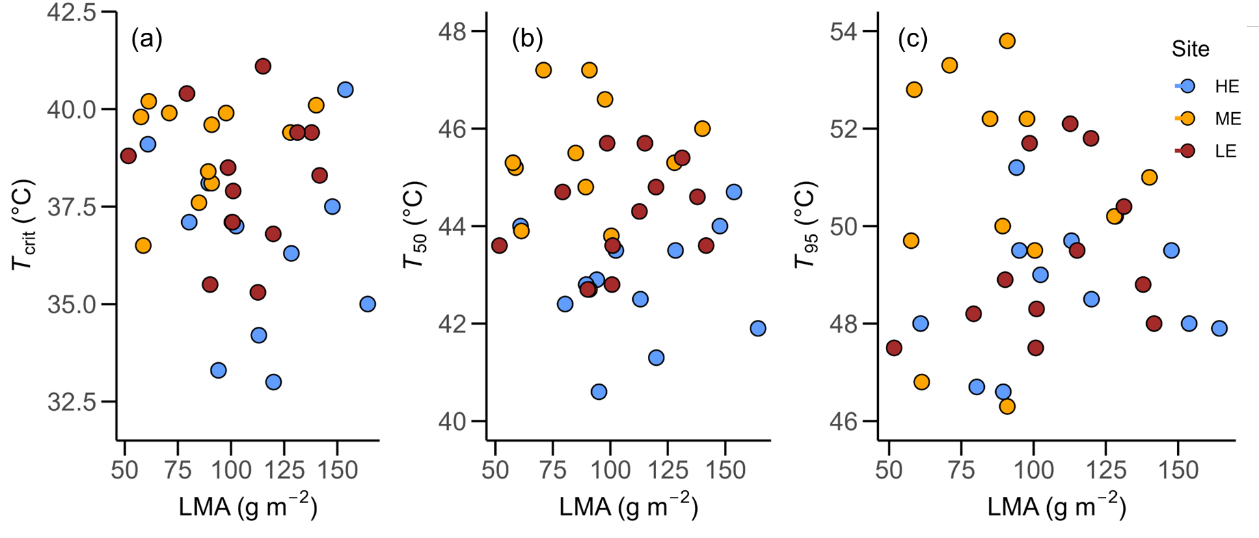


**Figure S9.**  Relationship between leaf mass per area (LMA) and the heat tolerance thresholds (a) *T*_crit_, (b) *T*_50_ and (c) *T*_95_. Data points represent the mean value for each species at each site. Different colours indicate different sites (blue: HE, high elevation site; yellow: ME, mid-elevation site; red: LE, low-elevation site).


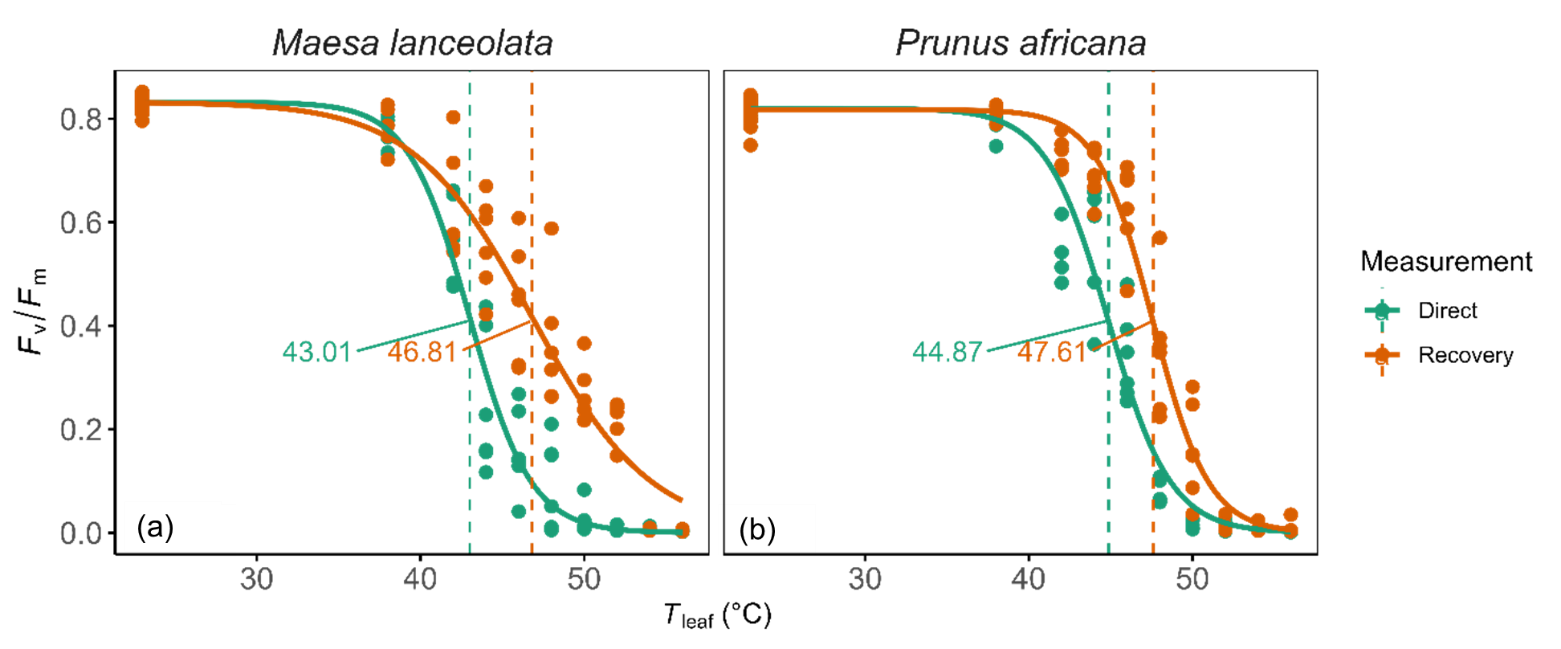


**Figure S10.** Effects of a 2-min heat pulse on the quantum yield of photosystem II (*F*_v_/*F*_m_) in (a) *Maesa lanceolata* and (b) *Prunus africana* recorded directly after heat treatment (green), or 24 h after heat treatment (orange). The dashed line represents *T*_50_.


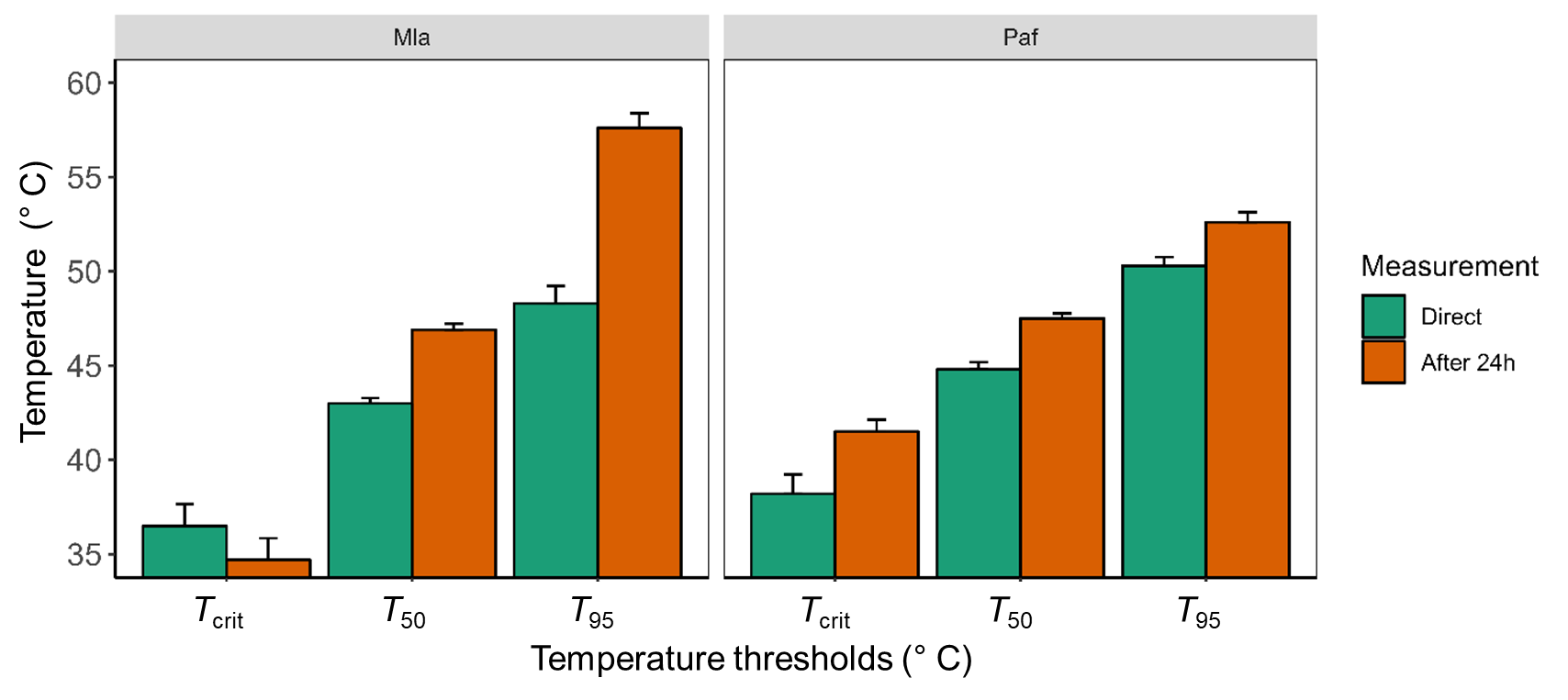


**Figure S11.** Variation in breakpoint temperature (*T*_crit_, °C) and the temperatures at which the F_v_/F_m_ is reduced by 50% (*T*_50_, °C) and 95% (*T*_95_, °C) compared to non-heat stressed conditions for *Maesa lanceolata* (green) and *Prunus africana* (orange). Error bars represent standard errors of means.


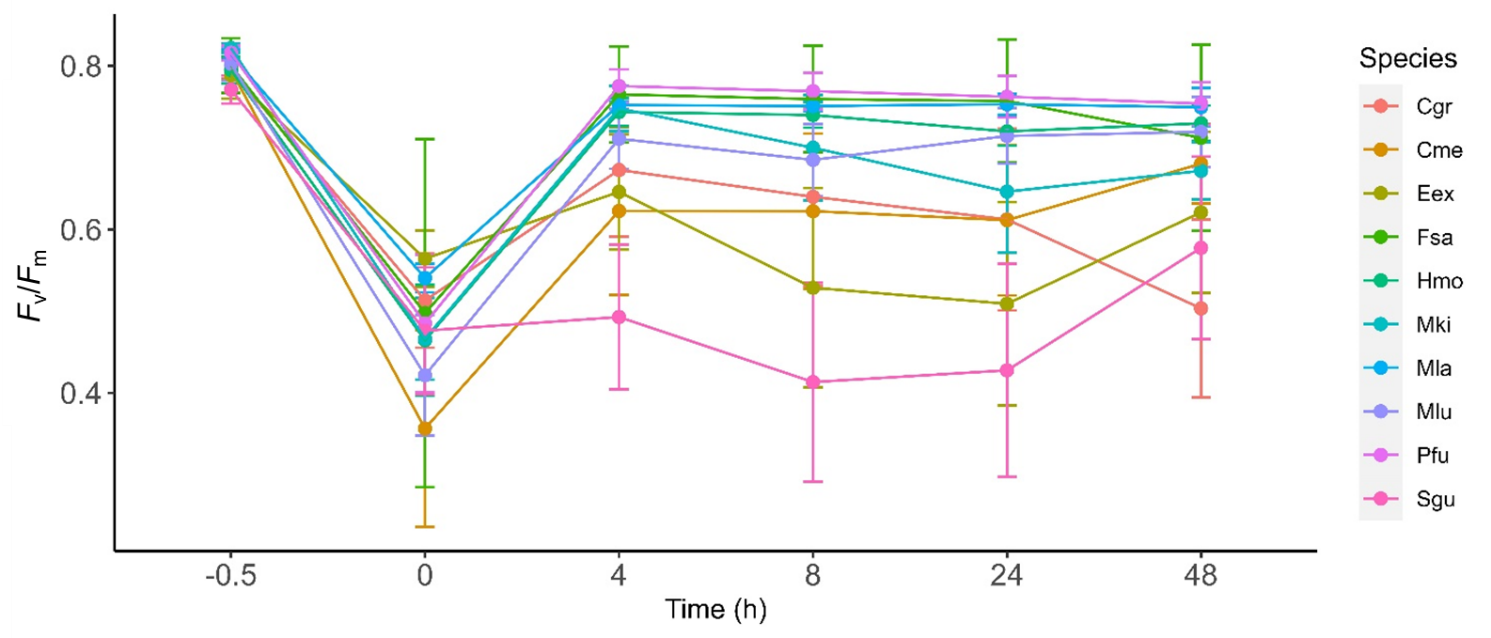


**Figure S12.** Temporal recovery of dark-adapted *F*_v_/*F*_m_ in studied species at the ME site over 48 hours post-heat exposure. The leaves were exposed to heat pulses corresponding to earlier recordings of species-specific *T*_50_ values, shown in Figure S1. The heat pulse was administered at 0 h. Species abbreviations are defined in Table 1. Error bars represent standard errors of means.
